# Drivers of partially automated vehicles are blamed for crashes that they cannot reasonably avoid

**DOI:** 10.1038/s41598-022-19876-0

**Published:** 2022-09-28

**Authors:** Niek Beckers, Luciano Cavalcante Siebert, Merijn Bruijnes, Catholijn Jonker, David Abbink

**Affiliations:** 1 AiTech, Delft University of Technology, Delft, Netherlands; 2 Cognitive Robotics, Faculty of Mechanical, Maritime, and Material Engineering, Delft University of Technology, Delft, Netherlands; 3 Interactive Intelligence, Faculty of Electrical Engineering, Mathematics and Computer Science, Delft University of Technology, Delft, Netherlands; 4 Public Governance and Management, Faculty of Law Economics and Governance, Utrecht University, Utrecht, Netherlands

**Keywords:** Human behaviour, Mechanical engineering

## Abstract

People seem to hold the human driver to be primarily responsible when their partially automated vehicle crashes, yet is this reasonable? While the driver is often required to immediately take over from the automation when it fails, placing such high expectations on the driver to remain vigilant in partially automated driving is unreasonable. Drivers show difficulties in taking over control when needed immediately, potentially resulting in dangerous situations. From a normative perspective, it would be reasonable to consider the impact of automation on the driver’s ability to take over control when attributing responsibility for a crash. We, therefore, analyzed whether the public indeed considers driver ability when attributing responsibility to the driver, the vehicle, and its manufacturer. Participants blamed the driver primarily, even though they recognized the driver’s decreased ability to avoid the crash. These results portend undesirable situations in which users of partially driving automation are the ones held responsible, which may be unreasonable due to the detrimental impact of driving automation on human drivers. Lastly, the outcome signals that public awareness of such human-factors issues with automated driving should be improved.

Self-driving vehicles are improving rapidly, yet they occasionally fail with potential severe consequences: from near-misses, to crashes resulting in damage, injury, or even loss of life^1–3^. While studies investigated the public’s opinion on the ethical principles that should guide the behavior of self-driving vehicles in critical situations such as accidents^4^, more recent efforts investigated the public’s opinion on how responsibility should be attributed when accidents with self-driving vehicles occur^5–9^. Because many stakeholders are involved, e.g. the driver, the automated vehicle, and its manufacturer^5,10^, assessing who is responsible when a crash occurs in automated driving and whether that responsibility attribution is reasonable is a complex problem. Understanding how the public would attribute responsibility is important, as it may shape vehicle design and legislation. In this work we focus on a specific aspect of responsibility, namely culpability (also referred to as blameworthiness), which assesses whether someone’s behavior deserves to be blamed or considered responsible for the accident^10,11^.

For manual driving and *fully* autonomous driving the public’s responsibility attribution seems relatively clear-cut: the driver of a non-automated vehicle is blamed in the event of a crash without mitigating circumstances^6–9,12^, whereas the manufacturer is blamed when a crash with a fully autonomous vehicle occurs^7–9,12^. The question of culpability, i.e. if blame is deserved, becomes more complicated for *partially* automated vehicles. These vehicles are not autonomous but take over control of driving tasks from the human driver for long periods. As a result, the driver’s role shifts from being directly in control to being an out-of-the-loop supervisor of the automation (e.g,^13,14^). This type of partial automation is dominant in the current automated vehicle market. However, such automation is still brittle and can fail unexpectedly^15^. Then, the automation trades control authority to the human. This unexpected control transfer has been shown to contribute to, or even lead to, accidents (e.g.,^16–20^).

Manufacturers of partially automated vehicles currently assign liability—i.e., legal responsibility—to the human driver by requiring them to remain vigilant and ready to take over control when requested at any time through their terms of use^21^. The general public shares this view that a driver of a partially automated vehicle is required to always be ready to take over: people blame the driver more than the automation when a crash occurs^8,9^. Similarly, Awad et al.^6^ found that humans are blamed more than the automation when both fail to avoid a crash. In other words, when the human and the automation make the same mistake, the human driver is blamed more. However, is blaming the driver primarily in these situations reasonable?

While the driver technically has the means to take over control of the vehicle—e.g., through grabbing the steering wheel or pushing an override button—a key element in culpability attribution is the extent to which the human driver is able to appropriately act and avoid the crash at the moment they were required to^10,11^. Indeed, scientists argue that the extent of a driver’s responsibility when interacting with automation, such as driving a partially automated vehicle, depends on to what degree they were able to control the system at that moment^22,23^. We define ability as the extent to having the competence, skill, and the opportunity (e.g., in time) to execute control including perception, action selection, and action following^22^. In this context, we find a critical gap in the aforementioned studies on blame attribution in partially automated driving^6,8,9,12^ as they did not explicitly consider whether the human driver was able to control the outcome.

Indeed, taking the driver’s ability into account when assigning culpability is important, as the design of automated vehicles that require drivers to supervise the automation can lead to significant driver-related issues including complacency, skill degradation, and loss of situation awareness (see^24^ for an overview). Asking a driver to supervise for prolonged periods drastically impacts their ability to take back control, and quickly and appropriately respond to unexpected situations^16–20,24–27^. A prominent cause is the loss of awareness of the environment and of the automated vehicle’s functioning^28,29^. Regaining this situation awareness requires time that may not be available given the time-critical nature of unexpected automation failures, hampering the driver’s ability to appropriately respond^16,18,27,29^.

These issues are exacerbated by the fact that humans do not excel at remaining vigilant even for short periods when supervising automation, exemplified by the fact that drivers tend to engage in undesirable non-driving related activities, such as mobile phone use^24,29^. On the one hand, drivers can lose situation awareness due to engaging in non-driving related tasks, such as using the vehicle’s entertainment system^28,30^. On the other hand, loss of situation awareness can also occur unintentionally: drivers’ minds tend to wander off when the driving tasks are monotonous^26,31,32^ as is often the case when supervising automation^33^. It is, therefore, important to consider the source of distraction in automated vehicles to assess culpability^34^. Moral judgment depends on the intention of an action; deciding to perform an action leading to negative consequences is blamed more than not deciding to do any action^12,35,36^. Distractions that result from intentionally deciding to do something non-driving related such as using the entertainment system might be considered more culpable than distraction due to unintentional behavior (e.g., the driver’s mind wandering off)^35–37^.

Taken together, there seems to be a mismatch between the public’s attribution of blame and what the human factors literature deems as blameworthy. Specifically, a gap exists between what is required from the driver when using an automated vehicle and what can be reasonable expected from them, posing a challenge to attribute culpability when a crash occurs^10^. In this paper we investigate culpability by assessing how information about a driver’s ability affects the public’s attribution of blame to the driver, vehicle, and manufacturer in situations where a crash occurred after a partially automated vehicle required the driver to suddenly take over control. We also investigate the reasons provided by participants for attributing blame and whether we can see a shift in blame attribution among the actors for different circumstances.

We used an online vignette study in which we asked our participants (N=250) to indicate to what extent the driver, the automated vehicle, and the vehicle’s manufacturer is considered responsible for a crash in different scenarios. We asked participants broadly on responsibility attribution to incorporate participants’ perspectives on legal, causal, moral, and role responsibility^11^ and asked participants to provide a textual motivation to their answers. The hypothetical scenarios contain realistic situations and descriptions of human driver behaviors based on empirical observations from human factors literature in real-world partially-automated driving (e.g.,^17,24^). The scenarios contain descriptions of the driver’s level of distraction that result from supervising the vehicle for a prolonged period to manipulate the perceived driver’s ability (following the presented human factors literature^17,22,24,29^), see Table 1 and Fig. 1. Drivers were either not distracted, distracted for a short period (in the order of seconds), or distracted for a long period (order of minutes). For the distracted scenarios, we varied whether the driver’s distraction was caused by intentionally engaging in a secondary task (e.g., using the vehicle’s entertainment system) or due to their minds wandering off unintentionally (e.g., thinking about dinner). In all scenarios, the automated vehicle is initially performing all the driving tasks for a long period of time successfully until a time-critical road situation occurs and the driver is requested to take over. The driver fails to take control and a crash occurs. Table 1 summarizes the scenario descriptions and the full vignettes can be found in the Supplementary methods.

Participants were randomly assigned to only one of the five scenarios resulting in fifty participants per scenario. We asked participants to rate the driver’s *level of situation awareness* and *ability to intervene* on 100-point scales to check whether the distraction descriptions resulted in the hypothesized impact on situation awareness and subsequently ability to take control and avoid the crash. Participants then assigned responsibility to the three involved actors: the driver, the automated vehicle, and the vehicle’s manufacturer on a 100-point scale. These ratings were analyzed with a moderated mediation regression model^38^, with awareness and control ability as mediators and actor and source of distraction as moderators (see Fig. 6). Participants’ motivations were analyzed through thematic analysis (see Methods for more information).

**Table 1:** Scenario descriptions.

	Distraction level	Source of distraction	Driver behavior description
1	Not distracted	–	“The driver stays focused on supervising the vehicle. As a result, the driver is paying full attention to the vehicle and the road”
2	Short distraction	Intentional	“The driver decides to look for a new podcast on the vehicle’s entertainment system. As a result, the driver is not paying attention to the vehicle and the road for a few seconds”
3	Short distraction	Unintentional	“The driver’s mind wanders off a bit on what to have for dinner. As a result, the driver is not actively paying attention to the vehicle and the road for a few seconds”
4	Long distraction	Intentional	“The driver decides to read news articles on the vehicle’s entertainment system. As a result, the driver is not paying attention to the vehicle and the road for a few minutes”
5	Long distraction	Unintentional	“The driver’s mind completely wanders off to day-dream about holiday plans. As a result, the driver is not actively paying attention to the vehicle and the road for a few seconds”

The automated vehicle was performing all the driving-related task successfully for an extended period of time before the crash occurred in each scenario. The driver’s behavior is varied per scenario. The driver and automated vehicle encounters an unknown situation and requests the driver to take over immediately. The driver fails to take over control and a crash occurs. Figure 1 shows two examples of the vignette visuals.

**Figure 1:**
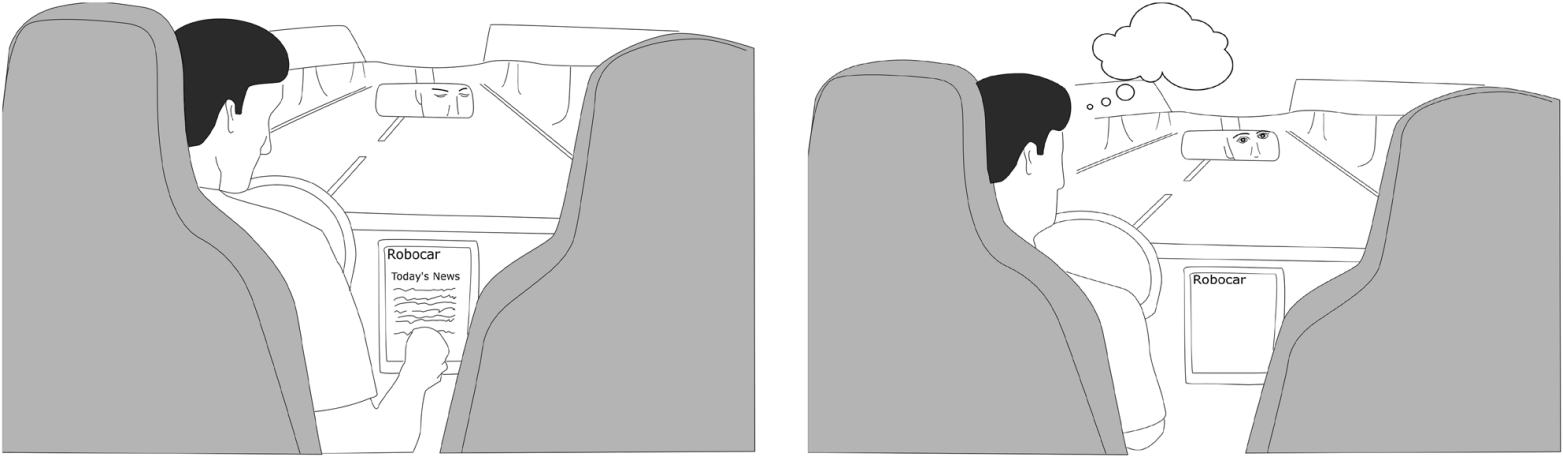
Two example vignette visuals of (left) an intentionally distracted driver engaging with the vehicle’s entertainment center and (right) an unintentionally distracted driver whose mind is wandering. See the Supplementary methods for all vignettes.

## Results

**Figure 2:**
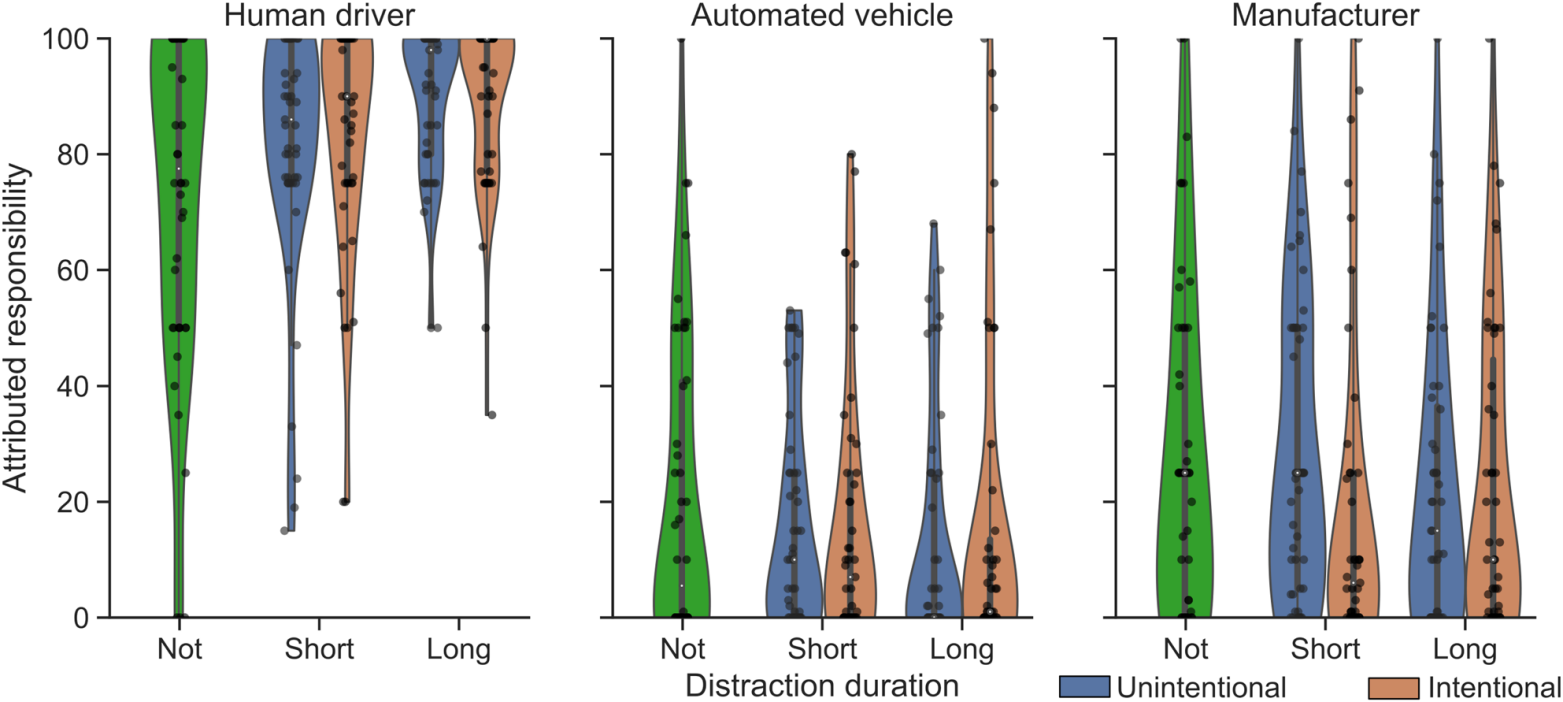
Responsibility attributed to each actor by the participants for all factor levels (distraction and cause of distraction). Data are visualized using violin plots, box plots, and individual data points. Cause of distraction was only varied within the distracted factor levels.

**Figure 3:**
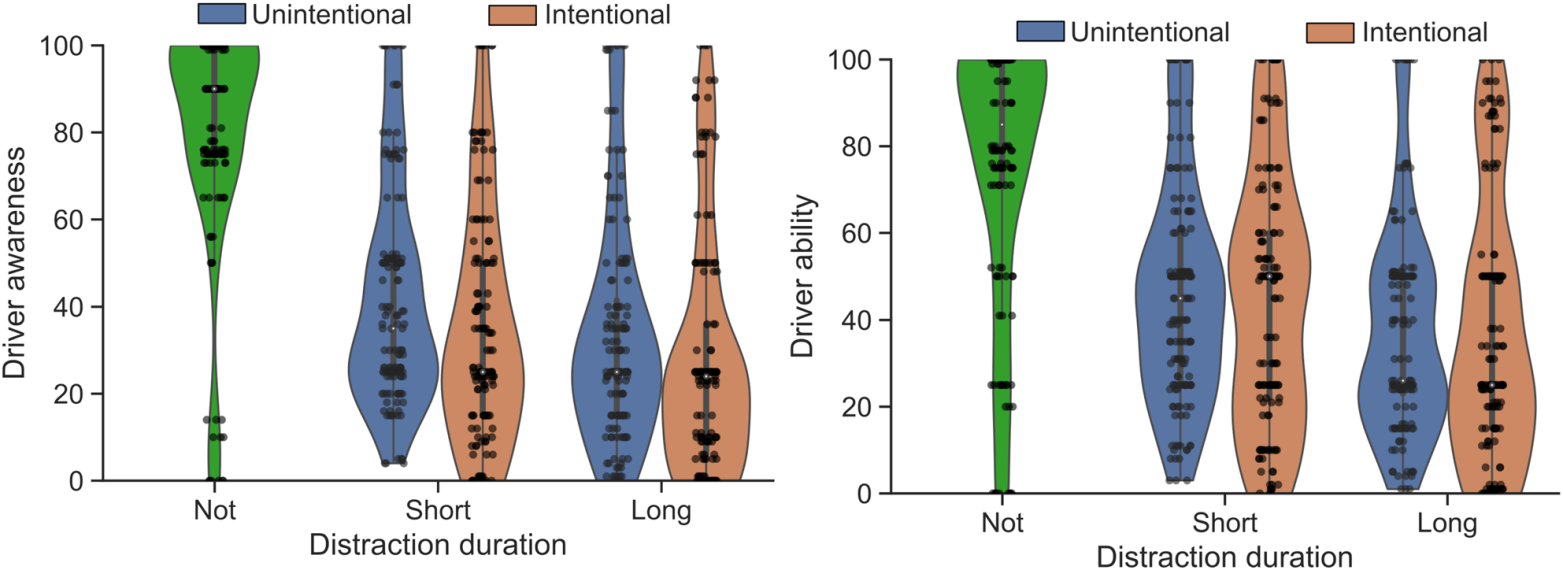
Driver’s situation awareness and ability to take control as perceived by the participants per distraction level and source of distraction. Data are visualized using box and violin plots.

The responsibility attributions per scenario (see Table 1) are shown in Fig. 2 and the driver awareness and ability are shown in Fig. 3. The model coefficients for the main effects are summarized in Table 2; the full model including interaction term coefficients can be found in the Supplementary table 1 and Supplementary figure 1.

The distraction level has a significant impact on the responsibility attributed to the driver. Participants blame the distracted driver (with short and long duration grouped) more compared to the not-distracted driver ($c_1=10.19$, 99% CI 0.33 to 21.50). The duration of distraction (comparing short versus long distraction duration) has no significant effect on the driver’s attributed responsibility ($c_2=3.78$, 99% CI − 0.50 to 7.60). Responsibility attribution did not depend on the source of distraction ($b_9=-4.36$, 99% CI − 10.26 to 1.94). Hence, the participants blamed the driver similarly regardless of whether the driver was distracted due to intentionally engaging in another task or unintentionally by their mind wandering off.

The overall responsibility attributed to the automated vehicle and its manufacturer was significantly lower compared to the driver ($b_2=-65.4$ CI − 80.0 to − 50.7 and $b_3=-55.61$, CI − 70.41, − 36.22, respectively). Interestingly, although the automated vehicle is not a human actor, participants still blamed it similarly compared to its manufacturer. In addition, the level of driver distraction and source of distraction did not moderate how participants attributed responsibility to the actors. In other words, we observed no shift in blame from the driver to the other actors, neither when the driver was more distracted, nor when the driver’s distraction was unintentional.

Participants rated the situation awareness of a distracted driver lower compared to a not-distracted driver ($a_{11}=-30.9$, 99% CI − 34.9 to − 25.8). A driver who is distracted for a longer period is also perceived to be less aware compared to a driver who is distracted for a short time ($a_{21}=-3.92$, 99% CI − 6.53 to − 1.19), see Fig. 3. Situation awareness is positively correlated to perceived ability to take control ($d = 0.45$, 99% CI 0.35 to 0.55). These results reflect that participants understand that distraction harms situation awareness, which in turn impacts the driver’s ability to take over control.

Although the distracted drivers were perceived to have less situation awareness and subsequent lower ability to take control, we found no significant impact on their attributed responsibility ($b_1=-0.01$, 99% CI − 0.2 to 0.17 and $b_2=0.07$, 99% CI − 0.07 to 0.23, respectively). Furthermore, the cause of distraction did not moderate the effect of the ability to take control on the driver’s blame ($b_{11}=0.04$, 99% CI − 0.09 to 0.16). This indicates that even if the decrease in the ability to take control is due to an unintentional distraction, the driver is blamed to a similar level compared to an intentionally distracted driver. Lastly, we found no interaction between ability and the type of actor on responsibility attribution ($b_7=-0.11$ and $b_8=-0.09$), suggesting that blame was not shifted to other actors when the driver was less able to intervene.

**Table 2:** Moderated mediation regression coefficient estimates and the 99% confidence intervals in brackets (bold represent significant effects) for the conceptual model in Fig. 6.

Outcome	Duration		Awareness	Ability	Actor		Cause	Intersect
	Not dist.–dist.	Short–long	$M_1$	$M_2$	Driver–AV	Driver–manuf.	*C*	
	$D_1$	$D_2$			$A_1$	$A_2$		
Awareness	$\mathbf {a_{11}= -30.9}$	$\mathbf {a_{21}=-3.9}$						$\mathbf {i_{M_1}=49.9}$
	**(− 34.9, − 25.8)**	**(− 6.5, − 1.2)**						**(47.2, 52.5)**
Ability	$\mathbf {a_{12}=-9.10}$	$a_{22}= -2.41$	$\mathbf {d=0.45}$					$\mathbf {i_{M_2}=30.0}$
	**(− 14.8, − 3.8)**	(− 5.11, 0.25)	**(0.35, 0.55)**					**(24.3, 36.2)**
Responsibility	$\mathbf {c_1=10.2}$	$c_2 = 3.78$	$b_1= -0.01$	$b_2=0.07$	$\mathbf {b_3=-65.4}$	$\mathbf {b_4=-55.6}$	$b_9= -4.36$	$\mathbf {i_R=78.4}$
	**(0.33, 21.5)**	(− 0.50 7.60)	(− 0.20, 0.17)	(− 0.07, 0.23)	**(− 80.0, − 50.7)**	**(− 70.4, − 36.2)**	(− 10.2, 1.9)	**(65.6, 89.2)**

Awareness, ability, and cause refer to situation awareness, ability to take control and successfully avoid the crash, and cause of the distraction, respectively. Note that we omit the (not-significant) interaction terms in this table; please see Supplementary table 1 for all model coefficients.

**Figure 4:**
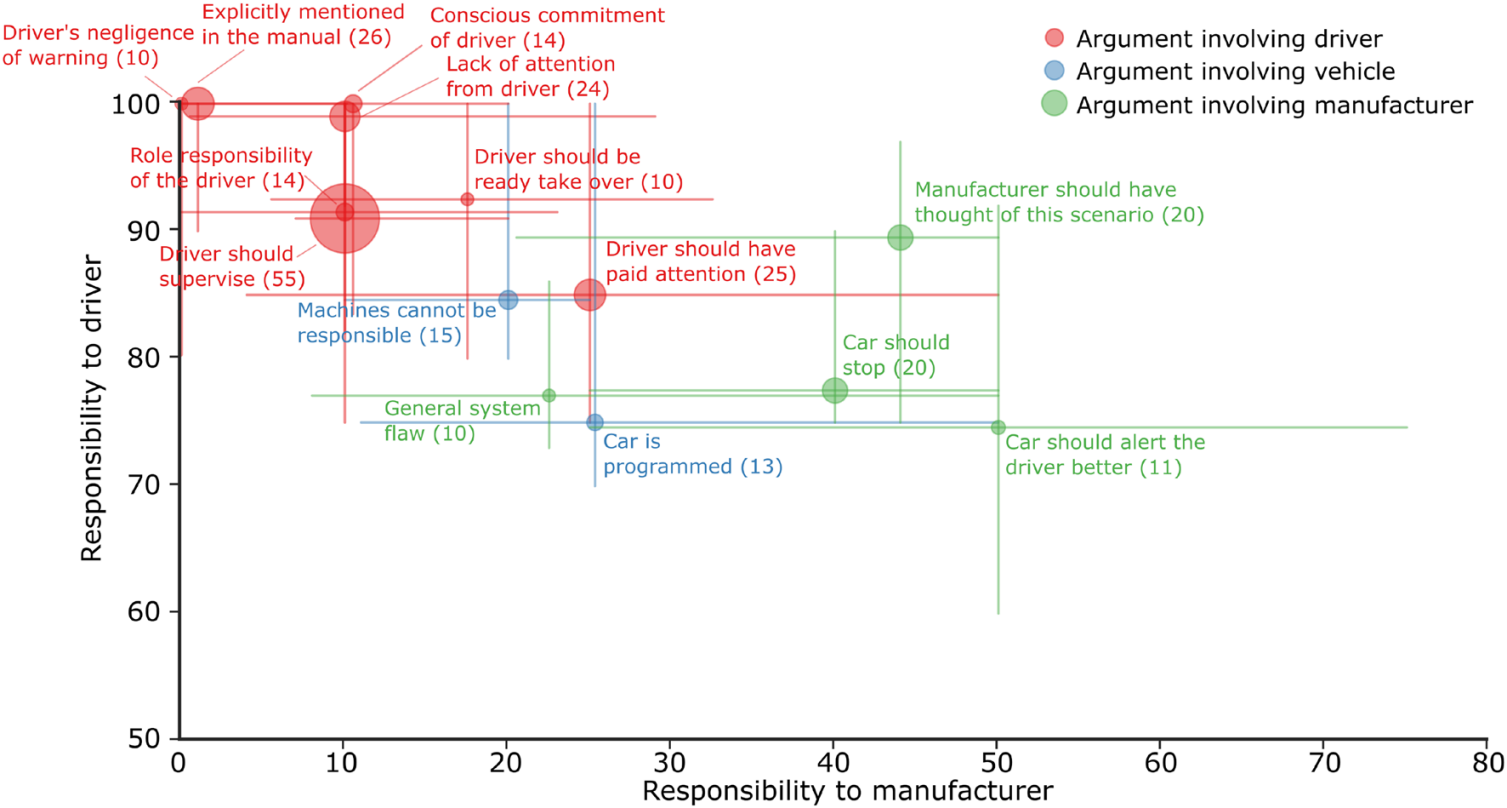
Median responsibility attribution to the driver and the manufacturer per code identified in the thematic analysis of the participants’ reasoning. Codes that were mentioned at least 10 times are visualized here for clarity (see Supplementary figures 17 and 18 for the other codes). The number of times the argument was made is included in brackets. The lines indicate 95% confidence interval of the median responsibility attributions to the driver and manufacturer for each code.

We analyzed participants’ motivations for their responsibility attribution through a thematic analysis. Two independent raters identified four themes, 17 codes (or labeled topics), and 52 sub-codes in the 238 participant comments without knowledge of the participants’ responsibility ratings. The themes sort the codes into arguments of explaining responsibility attribution toward the driver, the automated vehicle, the manufacturer, and the situation. Supplementary figures 14–17 show the codes, sub-codes, and a quote per sub-code for each theme. Grouping the participants’ responsibility ratings with the code corresponding by code revealed that responsibility attribution to the driver and manufacturer seems to be consistent with their reasoning, see Fig. 4. Participants who detail shortcomings or expectations of the driver attributed more responsibility to the driver and less to the manufacturer, while those who point out shortcomings of the manufacturer (including aspects related to the vehicle’s design) attributed less responsibility to the driver and more to the manufacturer. Participants that pointed out that the vehicle is a machine or a technical artifact and thus should not be blamed for any outcomes, in general, attributed less responsibility to both the driver and the manufacturer.

**Figure 5:**
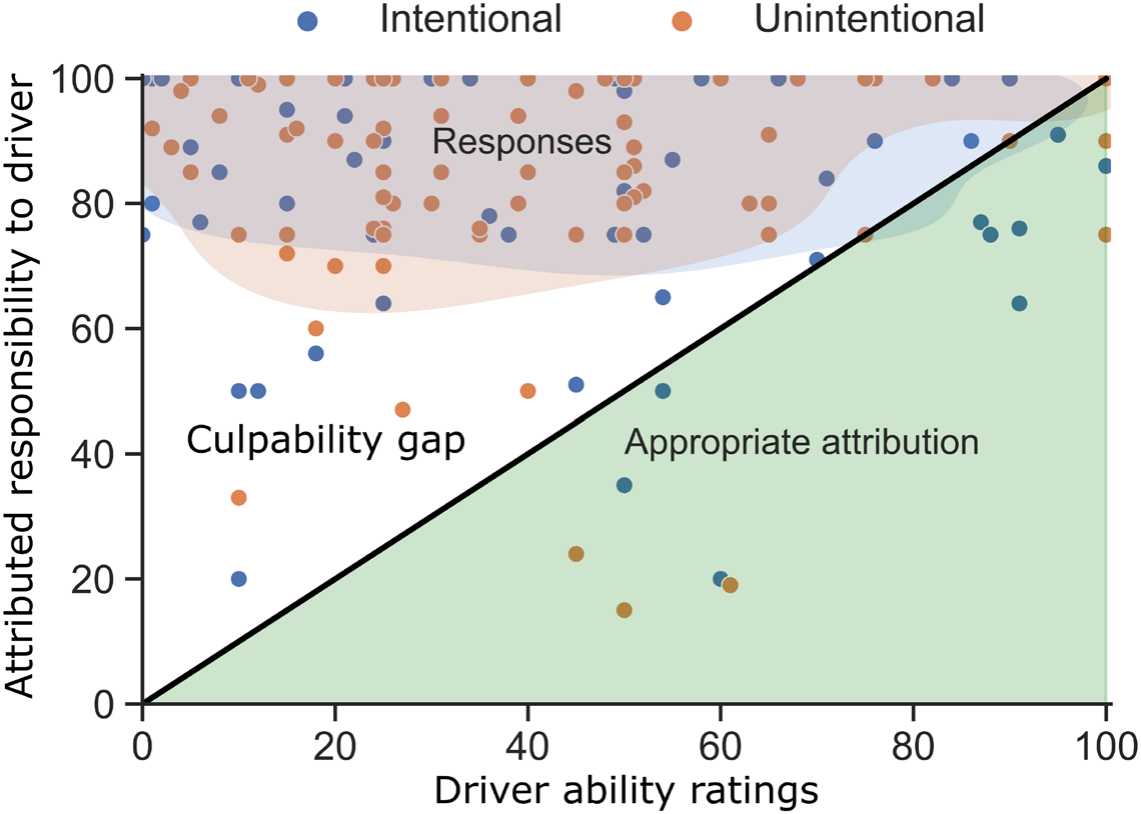
Attributed responsibility versus driver ability to take control and avoid the crash. The distribution of the responses for driver ability and corresponding attributed responsibility per participant are visualized using a kernel density estimate plot (Gaussian kernels, contour threshold at 0.25; e.g. 75% of the probability mass is indicated in the shaded areas) for the intentional and unintentional factor levels (short and long distraction factor levels pooled). The shaded areas represent 75% of the data probability mass per group. The black identity line is a qualitative representation of the normative expected attribution of responsibility given the driver’s ability to take control; attributed responsibility should be equal or lower to the driver’s control ability^22^.

Lastly, three main observations—(i) a distracted driver is perceived to be less able to take control and avoid the crash, (ii) the driver is held primarily responsible, and (iii) no blame is shifted to other actors—reveal a mismatch between participants’ responsibility attribution and whether this attribution is reasonable given the driver’s ability to take control of the automated vehicle and avoid the crash. The data seem to be at odds with a normative balance between ability and responsibility as argued by Flemisch et al.^22^. To illustrate this mismatch, Fig. 5 shows the quantitative responses of attributed responsibility versus the perceived ability combined with a qualitative representation of the normative balance between responsibility and ability. We only show the distracted conditions and differentiate between the intentional and unintentional causes of distraction. The identity line illustrates a qualitative normative balance between ability and attributed responsibility proposed by^22^; lower control ability should result in lower attributed responsibility. These results suggest that the public’s perception seems to ‘fall in a culpability gap’^10^, in particular when the driver’s ability to take control is low, which we will discuss in more detail below.

## Discussion

This study found that people who read about a crash involving a partially automated vehicle primarily blame the driver of the automated vehicle when a crash occurs, even when the driver’s ability to avoid the crash has deteriorated. Other studies also found that drivers, not the vehicle or its manufacturer, are primarily blamed in partially automated vehicles in which both driver and automated vehicle fail to avoid the crash^6–8,12^. What is surprising in the current study is that the participants acknowledged that the driver’s situation awareness and ability to intervene were impacted, yet this did not change their responsibility attribution as we expected. Our normative assumption was that drivers’ ability and responsibility would be balanced. This is particularly interesting for the cases when the impacted ability was due to an unintentional cause (mind wandering).

The ability to control an outcome is an important condition for culpability, which assesses whether someone’s behavior deserves to be blamed or considered responsible for a crash^39,40^. Indeed, studies found that when a driver has no ability to override a fully autonomous vehicle—e.g., no switch, button, steering wheel, or pedals—the general public shifts responsibility for a crash to the vehicle or its manufacturer instead of the driver compared to when a driver was manually driving the car^7,8,12,41^. However, when the driver has the means to override the automation, which is likely to remain in vehicles with increasing levels of automation for the foreseeable future, both the current study and another study^8^ found that drivers are held the most responsibility similar to when manually driving the vehicle. Uniquely, we found no effect of impacted ability on responsibility attribution. Only a few participants refer to the impacted driver ability by mentioning the limitations in human drivers, such as easily getting distracted and limited reaction speed; these participants attribute less responsibility to the driver.

The fact that drivers are mostly blamed could be due to the ‘foreseeability’ involved in partially automated vehicles—the driver should anticipate that the automation could fail^8,36^. Most of our participants’ arguments reflect this notion: among others, participants expected the driver to supervise and not get distracted, as well as that the driver made a voluntary choice and commitment when buying or driving a partially automated vehicle. Other arguments refer to the manual of the automated vehicle; the drivers should be aware of what is expected of them. Combined with the responsibility and ability ratings, this suggests that the majority of participants base their opinion using normative arguments against the driver: the driver committed to using an automated vehicle, and they failed to use it properly (e.g., they did not supervise the vehicle as they were supposed to).

Indeed, the public expects drivers to remain vigilant and supervise the automated vehicle at all times, yet we know this is an unreasonable demand for a human driver; even highly-trained pilots struggle with supervising autopilot systems for prolonged periods^13,14^. Driving automation has consistently been shown to impact driver vigilance and the ability to successfully take control, in particular in time-critical scenarios, which can happen without the driver’s awareness (e.g.,^24–27,29^). Following Flemisch et al.^22^, we argue that the responsibility attributed to a driver should be consistent with their ability to control the automated vehicle. If that ability is impacted by using the automation, responsibility should shift from the driver to the automation (or by proxy, its manufacturer), which raises the question whether our participants’ ratings are reasonable. Note that we only described typical behavior that occurs when driving with automated vehicles; we did not provide the participants with the aforementioned information about the known challenges of driving automation. It is an open question whether this will lead to shift in responsibility attribution.

The imbalance between these human-factor-related challenges with automation regarding driver ability and the participant’s responsibility attributions reveal a culpability gap^10^ (visualized in Fig. 5). In this culpability gap, responsibility is not reasonably distributed over the involved human agents; the driver receives most blame, yet this may be unreasonable given their impacted ability to change the outcome. The question is then what steps are needed for a reasonable distribution of responsibility to close this gap. The findings of this work have implications. In terms of public discourse, based on the participants’ arguments, it seems that the majority of our participants do not consider the aforementioned human-centered challenges of automated driving in their responsibility attribution. This could be an indication that humans are not aware of these effects of automation, which could lead to ‘unwitting omissions’^42^. Drivers are unaware of the impact of automated driving on their ability to perform the required driving tasks should they need to, yet they are still considered to be responsible by their peers. Providing public information about the driver-centered challenges associated with automated driving could be helpful, as well as driver training, but it remains to be seen whether this changes public responsibility attribution.

The public’s opinion on who is held responsible is important to consider, as public opinion on these matters can be expected to shape laws regarding automated vehicles^4,6,43^. Participants referred to the manual of use, indicating that participants agree with the manufacturer’s terms of use of the vehicle, most likely for liability reasons^21^. It is unclear whether these participants believe that these expectations of the manufacturer are reasonable. However, legal scholars argue that the state of the driver should be taken into account when evaluating liability after an accident with an automated vehicle, advocating that manufacturers should also bear part of the liability (e.g.,^44^). Similarly, regulatory commissions in the United Kingdom are recommending that automated vehicle users should not face regulatory sanctions when something goes wrong^45^.

It is an open question how public awareness and blame attribution may change when partially automated driving becomes more prevalent in our streets. Of our 250 participants, 137 never and 64 participants rarely drive a vehicle with partial automation (see Supplementary figure 13), showing that only a small portion of the participants have regular experience with partial driving automation. It is likely that with more exposure to partially automated vehicles, both when driving or participating in traffic with other automated vehicles, opinions may change.

This study has potential limitations. First, the wording of the scenarios can impact the participants’ responses. Although the descriptions were set up to be objective and accurate representations of the scenario, bias may still be present. To minimize bias, we used the terminology of user manuals of partially automated vehicles, accident reports involving partially automated vehicles, and human factors literature to create realistic and comprehensible scenarios. Essential concepts, such as ‘supervision’ or ‘taking over control’, are explained in more detail following descriptions in accident reports^1,2^ and wording used by manufacturers of partially automated vehicles in user manuals^21,46,47^. In addition, we acknowledge that describing underlying driver behavior (distraction) without explicitly stating its likely behavioral outcome (reduced ability to intervene) leaves room for interpretation. Similar studies described more explicit actions (e.g., “[the driver] decided to not intervene”^6^) or outcomes (e.g., “the semi-autonomous car hits the pedestrian”^8^), not the underlying behavior leading up to them (i.e., distraction in our case). We argue that providing information on the underlying behavior, which we based on human factors literature (e.g.,^24,26,28,29^), is essential and provides a more thorough account of the situation. Despite the potential issues in the wording of the descriptions, the ratings of awareness and ability, as well as the arguments for the responsibility rating, suggested that participants generally understood the scenarios. However, based on the ratings and arguments, it is an open question whether intentional and unintentional cause of the distraction is appropriately taken into consideration (see Supplementary table 2; participants used similar arguments for both intentional and unintentional scenarios).

Although we described potential behavior that has been observed in the real-world driving and situations that have occurred on the road, the participants know they are reading about hypothetical scenarios. It may reduce the psychological realism of the study, causing the responses to be different from what they would after reading about an actual event. Moreover, judgments do likely not occur using information solely provided in our scenarios but will be shaped by many factors beyond our control. People may have overly positive views of automated vehicle capabilities based on promises made by manufacturers, reports of accidents, or opinion pieces. These will influence the participants’ judgments.

It is a topic of debate whether or not failing to monitor automation and intervene when necessary is a typical ‘human error’ that should be remedied by policies^21,44,48,49^, more training and public education, and increased automation; or whether it is a symptom of inappropriate human-automation interaction design that should be remedied by human-centered design methodologies, for example through shared control^23^. Either way, we believe that such failures would still occur and a reasonable approach for responsibility attribution in such cases should be considered. We argue that the well-understood limitations in human abilities have to be accepted as they are, and should be used to realize appropriate attribution of responsibility to the driver, or the manufacturer of the vehicle (or by proxy the developers of the automated driving algorithms that control it) in case of accidents, by the general public as well as other stakeholders.

## Methods

**Figure 6:**
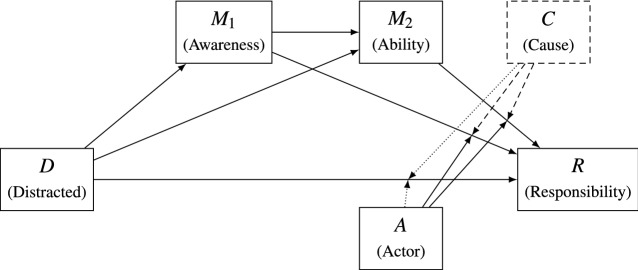
The conceptual model; the corresponding statistical model is shown in Supplementary Fig. 1.

We ask our participants (N=250) in an online vignette study to attribute responsibility in a scenario in which a human driver and their partially automated vehicle were involved in a crash. We assess the effect of driver distraction (denoted by *D*), source of distraction (*C*) on the responsibility *R* attributed to each actor (*A*). Participants also rated their perception of the driver’s situation awareness ($M_1$) and driver ability to take over control and avoid the crash ($M_2$). Figure 6 shows the conceptual model of the relations between these independent and dependent variables. We hypothesize that distraction will impact the driver’s situation awareness, which in turn affects the driver’s ability to take control and successfully intervene. The impact of driver ability on their attributed responsibility is analyzed.

Participants were recruited through the online crowd-worker platform Prolific to assure high-quality data^50^. We estimated the minimum required sample size to be 204 participants using an effect size $f^2=.1$ (between small and medium effect), $\alpha =.05$, power $(1-\beta )=.95$ and ten predictors (each predictor path, see Fig. 6) of which five are measured. Accommodating for expected attrition (i.e., failed attention checks) and available funds, we decided a-priori to recruit a total of 250 participants. Participants were paid for their time according to the platform’s norms. Participants were uniformly randomly assigned to one of the five scenarios. Participants could only take part in the study if they had a valid driver’s license. Twelve participants failed the attention check questions and were excluded from further analysis. The remaining participants are aged 18–76 years (median of 25 years) and 39% are female. After the main experiment, participants were asked about their general attitude toward driving automation, including trust in driving automation, and technology adoption to check whether attitudes toward driving automation could influence the results (see Supplementary figure 13). The experiment was carried out in accordance with the university’s guidelines and regulations. The Human Research Ethical Committee of Delft University of Technology approved the research under number 1277. We obtained informed consent from all participants.

### Scenarios

The scenarios are hypothetical, but are designed to contain realistic situations and descriptions of driver behaviors in real-world automated driving in human-factors literature^22,24–26,28,29^ and automated vehicle accident reports^1–3^. We describe the underlying behavior (distraction and cause of the distraction) that are shown to impact situation awareness and subsequent ability to take control rather than explicitly stating these factors (e.g., the driver was able to take control) to avoid biasing the participants. To check whether the descriptions of driver behavior were interpreted as intended, we asked participants to rate the degree of situation awareness and degree of ability based on the descriptions prior to attributing responsibility.

We created the scenarios to compare responsibility attribution for different levels of driver distraction: not distracted, short distracted (order of seconds), and long distracted (order of minutes). Loss of situation awareness can occur even over short periods of reduced vigilance i.e., when briefly distracted^29^, which we operationalized in the short distraction scenarios. The long distraction scenarios are used to include distractions that are typical when over-relying on automation, which is detrimental for vigilance and loss of situation awareness^28,29^. The cause of distraction was either intentional (actively engaging with the vehicles entertainment system) or unintentional (the driver’s mind wandering off). This resulted in five scenarios that are listed in Table 1. See the Supplementary methods for the full scenario descriptions.

### Metrics

We asked our participants to attribute responsibility to each of following three actors (denoted by *A*): the human driver (“Robyn”), the automated vehicle (“Robocar”), and the vehicle’s manufacturer (“Manufacturer”). We included the automated vehicle as an actor to explore the extent to which the public views automated vehicles as moral agents, following previous studies^7,8^. We measured attributed perceived responsibility for each actor (driver, automated vehicle, and manufacturer) on a 100-point scale ranging from ‘totally not’ (0) to ‘totally’ (100) by asking “To what extent is each actor responsible for the accident?”. The perceived extent of the driver’s situation awareness was assessed through the question “To what extent would Robyn be aware of the situation?” on a 100-point scale from ‘totally not aware’ to ‘totally aware’. Participants then gave their perception of the driver’s ability to take control (“Can Robyn take control to successfully deal with the situation?”), again on a 100-point scale from ‘totally not’ to ‘totally’. At the end of the questionnaire participants filled out questions regarding driving frequency, experience with driving automation, and attention checks whether they read the scenario correctly.

### Statistical analysis

The data was analyzed using a moderated mediation model shown in Fig. 6, in which situation awareness $M_1$ and ability $M_2$ are mediators , and cause of distraction *C* and actors *A* are moderators^38,51,52^. Because both distraction duration *D* and actor *A* are multi-categorical variables with three factor levels, we defined two contrasts per factor^38^, see Table 3. Contrast $D_1$ compares the not-distracted driver scenarios with the distracted driver scenarios (combining short and long distraction and pooling cause of distraction). Contrast $D_2$ compares the short and long distraction levels (cause is pooled). The actor groups are coded with respect to the human driver, resulting in two groups comparing human driver with automated vehicle (group $A_1$) and human driver with manufacturer (group $A_2$).

**Table 3:** Contrast groups for distraction and actor.

Label	Description	Coding		
*Distraction*		Not distracted	Short	Long
$D_1$	Not distracted versus distracted	− 1	1/2	1/2
$D_2$	Short versus long distracted	0	− 1	1
*Actor*		Robyn	Robocar	Manufacturer
$A_1$	Robyn versus Robocar	0	1	0
$A_2$	Robyn versus Manufacturer	0	0	1

The conceptual model in Fig. 6 translates into three linear equations:

1 $$\begin{aligned} M_1&= i_{M_1} + a_{11} D_1 + a_{21} D_2 + e_{M_1} \end{aligned}$$

2 $$\begin{aligned} M_2&= i_{M_2} + a_{12} D_1 + a_{22} D_2 + d M_1 + e_{M_2} \end{aligned}$$

3 $$\begin{aligned} R&= i_Y + c_1 D_1 + c_2 D_2 + b_1 M_1 + b_2 M_2 + b_3 A_1 + b_4 A_2 \nonumber \\&\quad + c_3 D_1 A_1 + c_4 D_1 A_2 + c_5 D_2 A_1 + c_6 D_2 A_2 + b_5 M_1 A_1 + b_6 M_1 A_2 + b_7 M_2 A_1 + b_8 M_2 A_2 \nonumber \\&\quad + b_9 C + b_{10} M_1 C + b_{11} M_2 C + b_{12} D_2 C + e_{R} \end{aligned}$$

The model coefficients for $M_1$ and $M_2$ are estimated using ordinary least-squares. Because *R* depends on between- and within-participant factors, *R* is a linear-mixed effect model that is fitted using a maximum log-likelihood method. In addition to estimating the model coefficients, we also calculated the indirect effects from *D* to *R* through $M_1$ and $M_2$ by multiplying the coefficients corresponding to the indirect path. The moderation effects of *A* and *C* on the indirect effects are analyzed using the approach outlined in^52^. We use strict 99% bootstrap confidence intervals using 10,000 samples for all coefficients and indirect effects; coefficients with confidence intervals that do not include zero are statistically significant^51^. We perform the analysis in two steps. First we analyze the model without source of distraction, including all distraction contrasts and actor groups. We then analyze the effect of source of distraction on perceived responsibility only including the distracted conditions (i.e., only considering contrast $D_2$).

### Thematic analysis

Participants’ answers to an open-ended question asking to explain the reasoning for their responsibility attribution were analyzed by two independent raters following the thematic analysis method outlined by Braun and Clarke^53^. Codes and subcodes were generated systematically by the independent raters and then collated into thematic maps and applied to the entire dataset to generate frequencies. The codes, subcodes, and thematic maps were finalized only after unanimous agreement was reached in a discussion between the raters and the first three authors.

## Supplementary Information

Supplementary Information.

## Data Availability

Data for all figures and tables are available at 10.4121/16652056.v1.

## Code availability

The software used for analysis and generating the figures is available at 10.4121/16652056.v1
