# Supplementary material for: Drivers of partially automated vehicles are blamed for crashes that they cannot reasonably avoid
Source: Sci Rep. 2022 Sep 28;12:16193. doi: 10.1038/s41598-022-19876-0 (PMC9519957; doi:10.1038/s41598-022-19876-0)
Supplement: Supplementary file 1 — Supplementary Information. [file 41598_2022_19876_MOESM1_ESM.pdf]

Drivers of partially automated vehicles are blamed for crashes that they cannot reasonably avoid Supplementary information

Niek Beckers[1,2,*,†], **Luciano Cavalcante Siebert**[1,3,†], **Merijn Bruijnes**[4,†], **Catholijn Jonker**[1,3], **and David Abbink**[1,2]

[1]AiTech, Delft University of Technology
[2]Cognitive Robotics, Faculty of Mechanical, Maritime, and Material Engineering, Delft University of Technology
[3]Interactive Intelligence, Faculty of Electrical Engineering, Mathematics and Computer Science, Delft University of Technology
[4]Public Governance and Management, Faculty of Law Economics and Governance, Utrecht University
[†]Co-first author

Statistical model and results

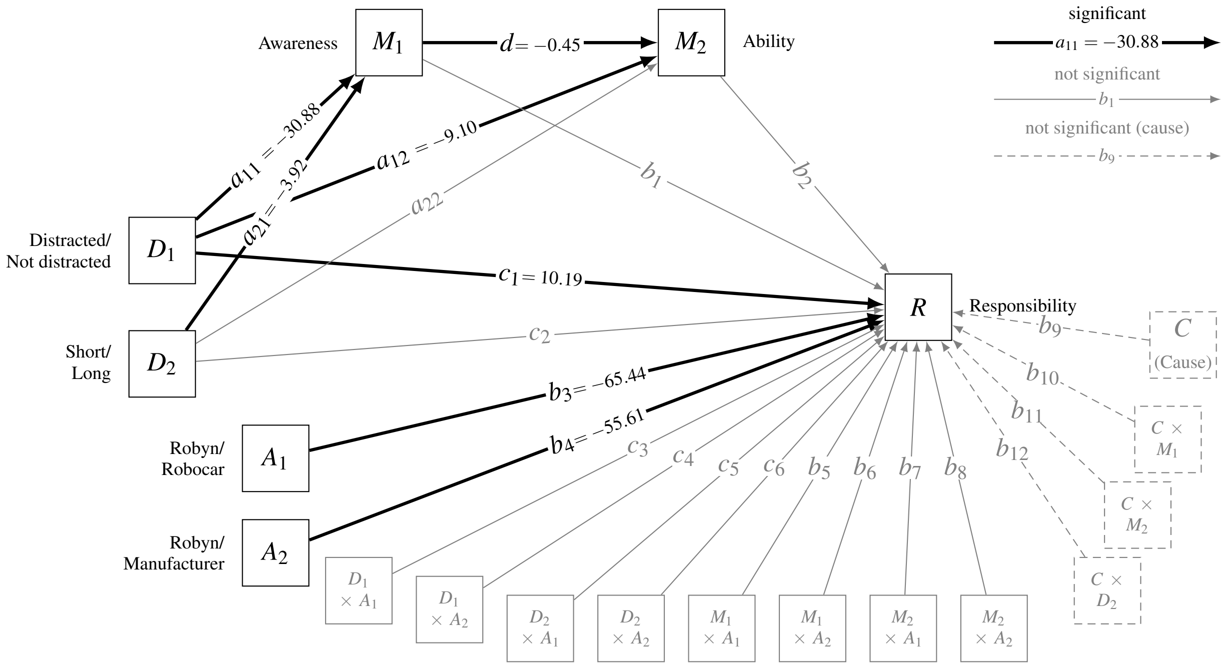

Supplementary figure 1. Statistical model including the statistically significant regression coefficient values.

Supplementary Table 1. All regression coefficients with confidence intervals (standard error in parentheses)

Outcome:		Awareness (M_1)			Ability (M_2)			Responsibility (R)	
		Coeff.	CI (99%)		Coeff.	CI (99%)		Coeff.	CI (99%)
Duration									
(D_1)	$\mathbf{a_{11}} \rightarrow$	**-30.88 (1.70)**	**-34.93, -25.82**	$\mathbf{a_{12}} \rightarrow$	**-9.10 (2.22)**	**-14.84, -3.81**	$\mathbf{c_1} \rightarrow$	**10.19 (4.11)**	**0.33, 21.50**
(D_2)	$\mathbf{a_{21}} \rightarrow$	**-3.92 (1.05)**	**-6.53, -1.19**	$a_{22} \rightarrow$	-2.41 (1.05)	-5.11, 0.25	$c_2 \rightarrow$	3.78 (1.60)	-0.50 7.60
Awareness (M_1)				$\mathbf{d} \rightarrow$	**0.45 (0.04)**	**0.35, 0.55**	$b_1 \rightarrow$	-0.01 (0.07)	-0.20, 0.17
Ability (M_2)							$b_2 \rightarrow$	0.071 (0.06)	-0.07, 0.23
Actor									
Robyn – Robocar (A_1)							$\mathbf{b_3} \rightarrow$	**-65.44 (5.98)**	**-80.00, -50.67**
Robyn – Manufacturer (A_2)							$\mathbf{b_4} \rightarrow$	**-55.61 (6.57)**	**-70.41, -36.22**
($D_1 \times A_1$)							$c_3 \rightarrow$	-11.02 (5.37)	-25.31, 2.21
($D_1 \times A_2$)							$c_5 \rightarrow$	-11.93 (6.00)	-27.20, 1.47
($D_2 \times A_1$)							$c_4 \rightarrow$	-4.03 (2.27)	-9.40, 2.12
($D_2 \times A_2$)							$c_6 \rightarrow$	-4.16 (2.42)	-10.27, 1.47
($M_1 \times A_1$)							$b_5 \rightarrow$	0.13 (0.09)	-0.11, 0.37
($M_1 \times A_2$)							$b_6 \rightarrow$	0.08 (1.00)	-0.16, 0.35
($M_2 \times A_1$)							$b_7 \rightarrow$	-0.11 (0.08)	-0.29, 0.09
($M_2 \times A_2$)							$b_8 \rightarrow$	-0.09 (0.09)	-0.34, 0.12
Cause of distraction									
(C)							$b_9 \rightarrow$	-4.36 (2.44)	-10.26, 1.94
($D_2 \times C$)							$c_7 \rightarrow$	1.76 (1.29)	-1.49, 5.02
($M_1 \times C$)							$b_{10} \rightarrow$	0.08 (0.05)	-0.07, 0.21
($M_2 \times C$)							$b_{11} \rightarrow$	0.04 (0.05)	-0.09, 0.16
Constant	$\mathbf{i_{M_1}} \rightarrow$	**49.9 (1.06)**	**47.17, 52.52**	$\mathbf{i_{M_2}} \rightarrow$	**29.98 (2.43)**	**24.32, 36.17**	$\mathbf{i_R} \rightarrow$	**78.39 (4.59)**	**65.56, 89.16**
		$R^2 = 0.3328$			$R^2 = 0.3491$			$R^2 = 0.6245$	

Study vignettes

Background of the scenario

All vignettes started with the following background description.

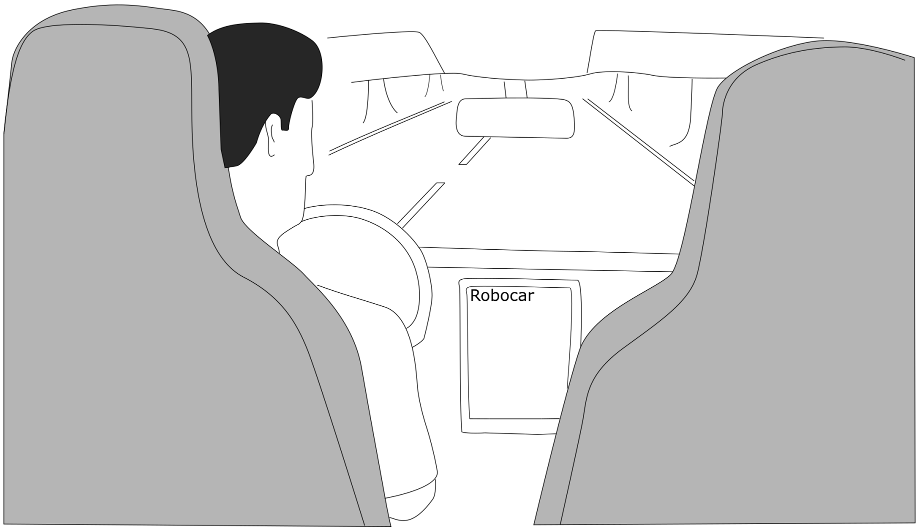

Background scenario

Robyn recently bought a new automated car, called Robocar. In Robocar's operating manual it says that Robocar can accelerate, brake, and steer the car by itself for long periods of time. "I love driving myself, but sometimes it is nice to have the car drive itself," says Robyn.

When the Robocar is in the automated driving mode, Robyn does not need to do any of the driving. Still, the manual states that Robyn has to supervise Robocar while it is driving. Supervising Robocar means that Robyn needs to be aware of what Robocar is doing and what happens on the road. Robocar can request Robyn to take control of the driving task when needed, for example when Robocar encounters a situation on the road that Robocar does not recognize or is not programmed to handle by itself. Taking over control of the driving task means that Robyn now has to steer, accelerate and brake the car alone, without Robocar.

Supplementary figure 2. Scenario background description used in the online vignette study. All participants read this background information before they read the separate vignettes depending on the experiment conditions they were assigned to (see below).

Vignettes for condition 1 (not distracted condition)

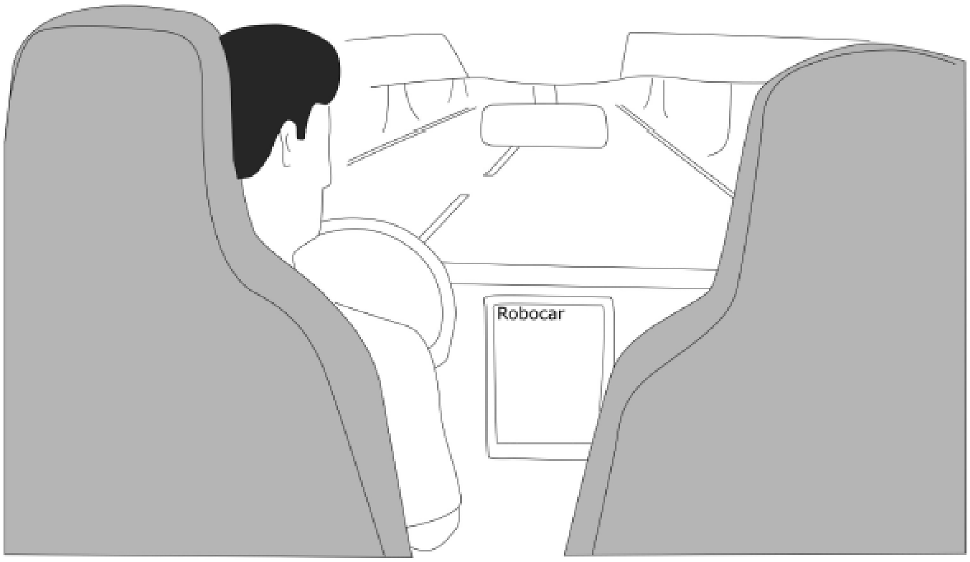

Robyn is travelling along a curvy two-lane road at the speed limit with no traffic. Robocar is in full control of all the driving tasks and Robyn's only task is to supervise Robocar.

The Robocar has been driving by itself for a long time. Robyn did not have to do any driving during this time. Robyn stays focused on supervising Robocar. Because of this, Robyn is paying full attention to Robocar and the road.

To what extent would Robyn be aware of the situation?

Totally not aware — Totally aware

0 25 50 75 100

Supplementary figure 3. Vignette 1 (not distracted condition). Participants first read the vignette with a description of the situation and driver distraction, and then rate the driver's awareness, ability and finally attributed blame to the three actors (human driver, automated vehicle, and manufacturer.

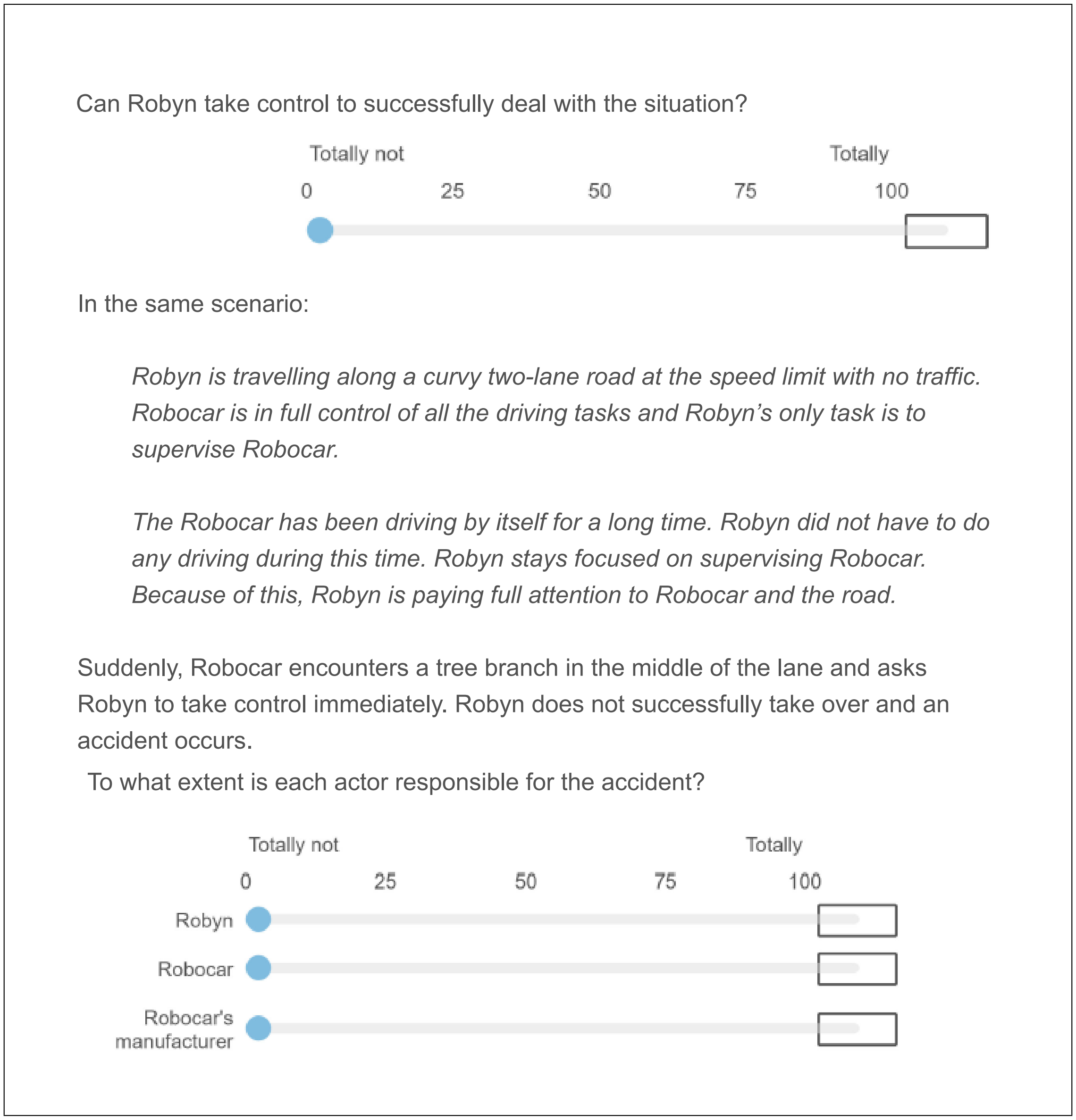

Supplementary figure 4. Vignette 1 (not distracted condition), continued. Participants first read the vignette with a description of the situation and driver distraction, and then rate the driver's awareness, ability and finally attributed blame to the three actors (human driver, automated vehicle, and manufacturer.

Vignettes for condition 2 (short distraction, unintentional distraction)

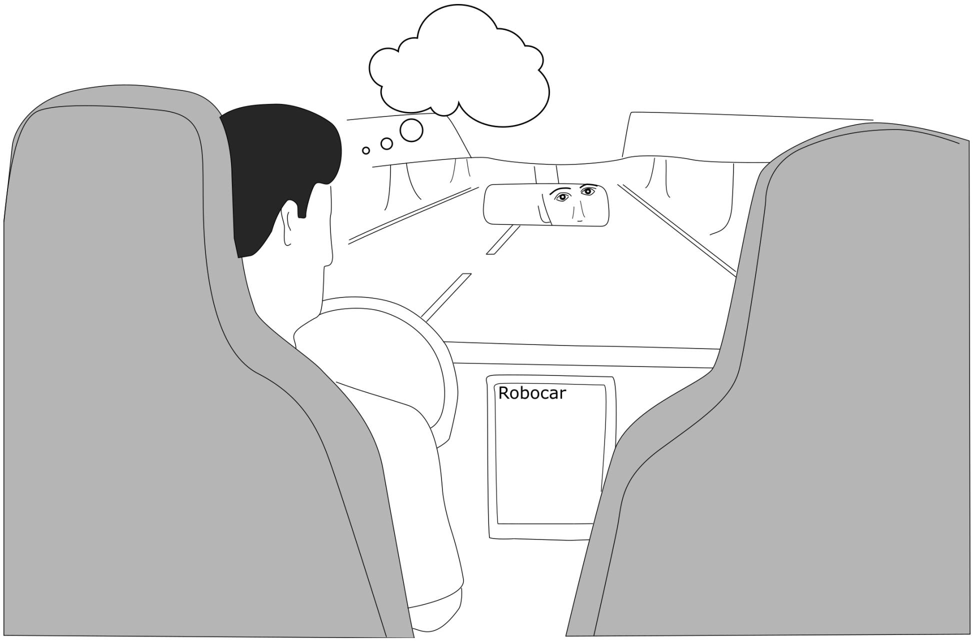

Consider this scenario:

> *Robyn is travelling along a curvy two-lane road at the speed limit with no traffic. Robocar is in full control of all the driving tasks and Robyn's only task is to supervise Robocar.*
>
> *Robocar has been driving by itself for a long time. Robyn did not have to do any driving during this time. So, Robyn's mind wanders off a bit on what to have for dinner. Because of this, Robyn is not actively paying attention to the Robocar and the road for a few seconds.*

Imagine that suddenly an unexpected situation happens on the road, for example a tree branch in the middle of the lane.

Supplementary figure 5. Vignette 2 (short distraction, unintentional distraction condition). Participants first read the vignette with a description of the situation and driver distraction, and then rate the driver's awareness, ability and finally attributed blame to the three actors (human driver, automated vehicle, and manufacturer.

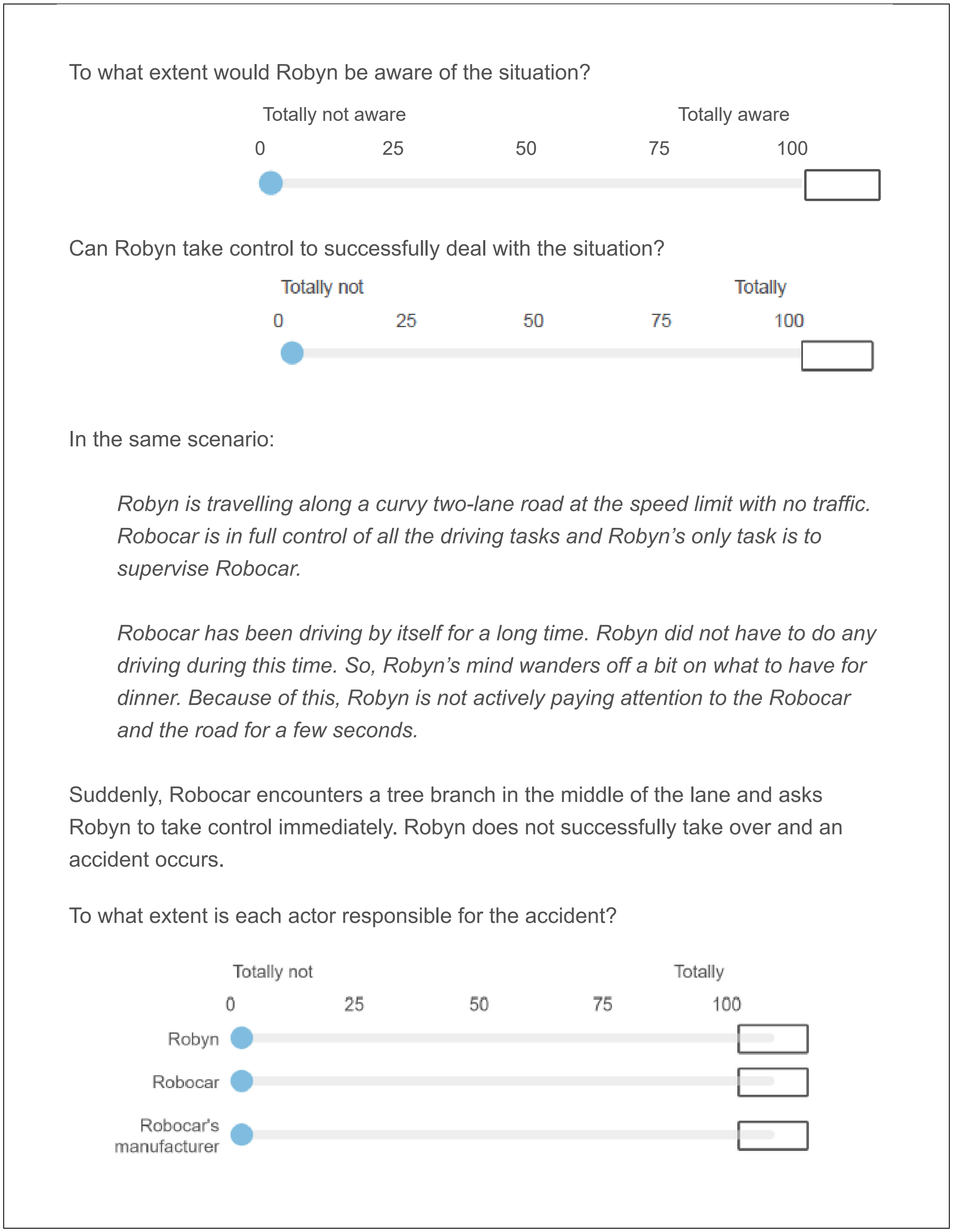
To what extent would Robyn be aware of the situation?

Totally not aware				Totally aware
0	25	50	75	100

Can Robyn take control to successfully deal with the situation?

Totally not				Totally
0	25	50	75	100

In the same scenario:

Robyn is travelling along a curvy two-lane road at the speed limit with no traffic. Robocar is in full control of all the driving tasks and Robyn's only task is to supervise Robocar.

Robocar has been driving by itself for a long time. Robyn did not have to do any driving during this time. So, Robyn's mind wanders off a bit on what to have for dinner. Because of this, Robyn is not actively paying attention to the Robocar and the road for a few seconds.

Suddenly, Robocar encounters a tree branch in the middle of the lane and asks Robyn to take control immediately. Robyn does not successfully take over and an accident occurs.

To what extent is each actor responsible for the accident?

	Totally not				Totally
	0	25	50	75	100
Robyn					
Robocar					
Robocar's manufacturer					

Supplementary figure 6. Vignette 2 (short distraction, unintentional distraction condition), continued. Participants first read the vignette with a description of the situation and driver distraction, and then rate the driver's awareness, ability and finally attributed blame to the three actors (human driver, automated vehicle, and manufacturer.

Vignette for condition 3: long distraction, unintentional distraction

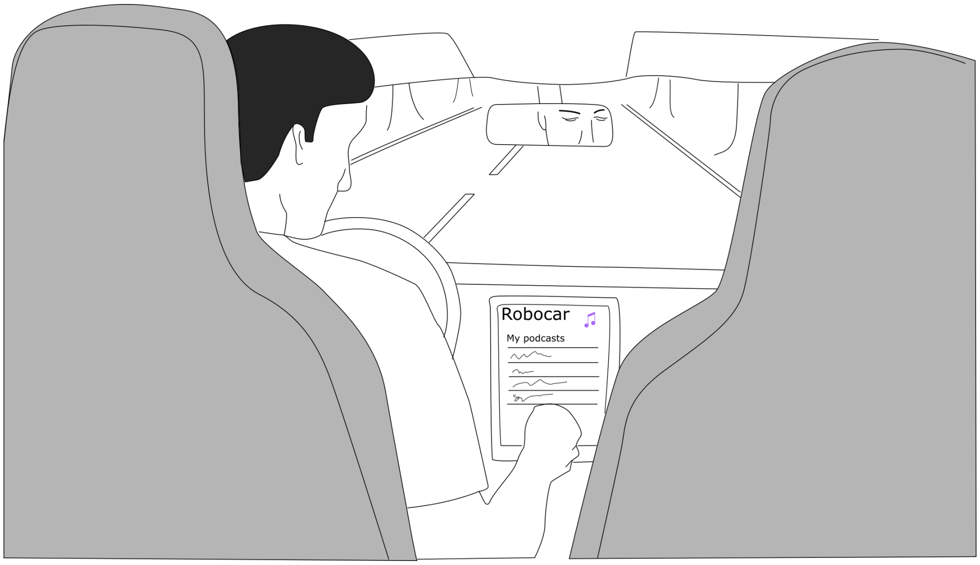

Consider this scenario:

> *Robyn is travelling along a curvy two-lane road at the speed limit with no traffic. Robocar is in full control of all the driving tasks and Robyn's only task is to supervise Robocar.*
>
> *Robocar has been driving by itself for a long time. Robyn did not have to do any driving during this time. So, Robyn decides to look for a new podcast on the vehicle's entertainment system. Because of this, Robyn is not paying attention to Robocar and the road for a few seconds.*

Imagine that suddenly an unexpected situation happens on the road, for example a tree branch in the middle of the lane .

To what extent would Robyn be aware of the situation?

Totally not aware | Totally aware

0 | 25 | 50 | 75 | 100

Supplementary figure 7. Vignette 3 (long distraction, unintentional distraction condition). Participants first read the vignette with a description of the situation and driver distraction, and then rate the driver's awareness, ability and finally attributed blame to the three actors (human driver, automated vehicle, and manufacturer.

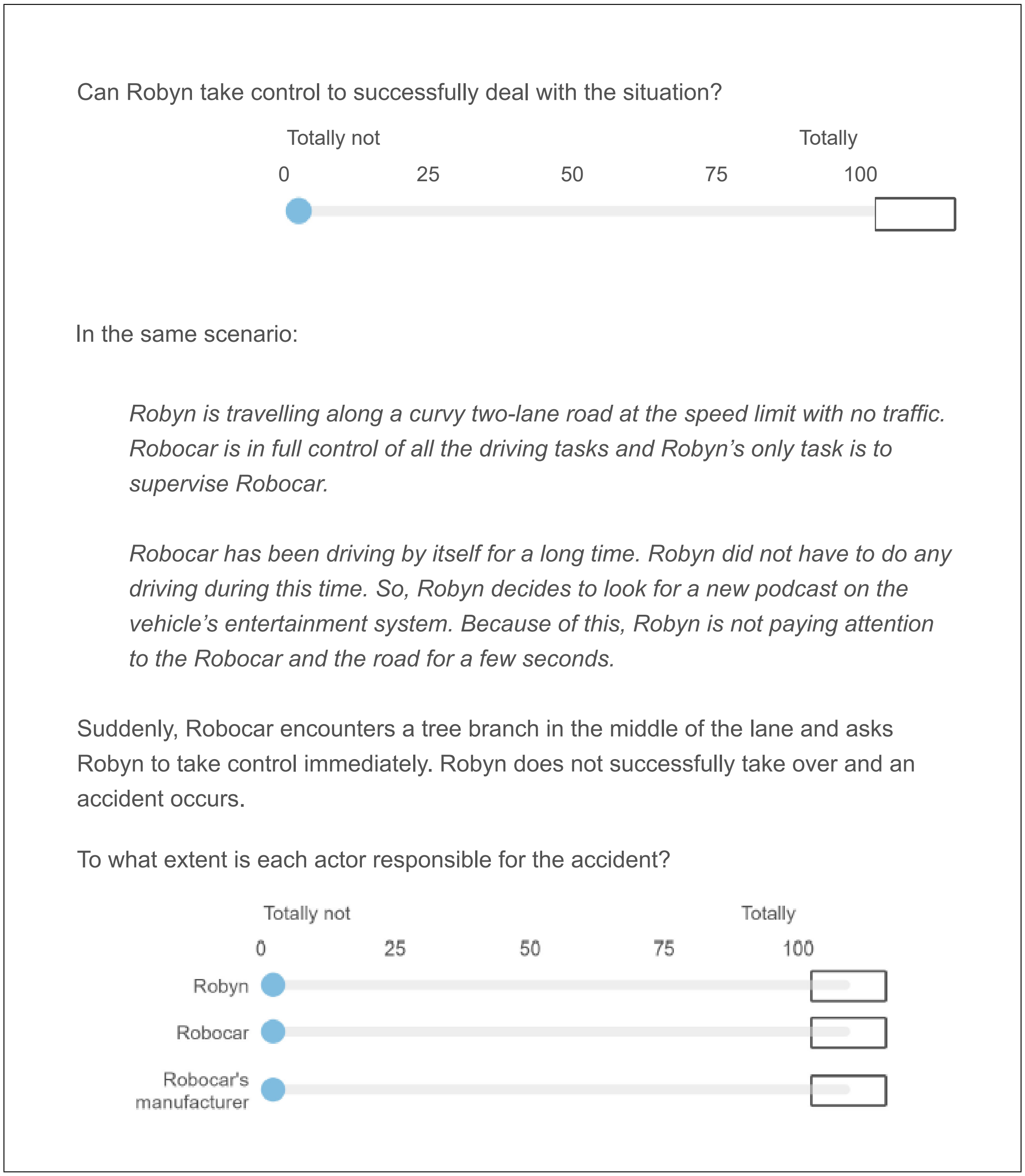

Can Robyn take control to successfully deal with the situation?

Totally not — Totally

0 25 50 75 100

In the same scenario:

Robyn is travelling along a curvy two-lane road at the speed limit with no traffic. Robocar is in full control of all the driving tasks and Robyn's only task is to supervise Robocar.

Robocar has been driving by itself for a long time. Robyn did not have to do any driving during this time. So, Robyn decides to look for a new podcast on the vehicle's entertainment system. Because of this, Robyn is not paying attention to the Robocar and the road for a few seconds.

Suddenly, Robocar encounters a tree branch in the middle of the lane and asks Robyn to take control immediately. Robyn does not successfully take over and an accident occurs.

To what extent is each actor responsible for the accident?

Totally not — Totally

0 25 50 75 100

Robyn

Robocar

Robocar's manufacturer

Supplementary figure 8. Vignette 3 (long distraction, unintentional distraction condition), continued. Participants first read the vignette with a description of the situation and driver distraction, and then rate the driver's awareness, ability and finally attributed blame to the three actors (human driver, automated vehicle, and manufacturer.

Vignette for condition 4: short distraction, intentional distraction

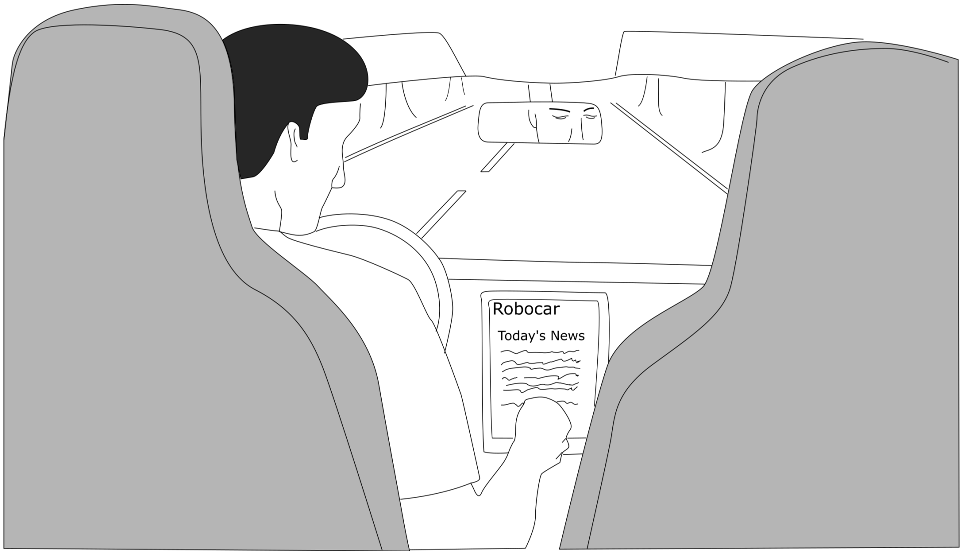

Consider this scenario:

> *Robyn is travelling along a curvy two-lane road at the speed limit with no traffic. Robocar is in full control of all the driving tasks and Robyn's only task is to supervise Robocar.*
>
> *The Robocar has been driving by itself for a long time. Robyn did not have to do any driving during this time. So, Robyn decides to read news articles on the vehicle's entertainment system. Because of this, Robyn is not paying attention to the Robocar and the road for a few minutes.*

Imagine that suddenly an unexpected situation happens on the road, for example a tree branch in the middle of the lane.

To what extent would Robyn be aware of the situation?

Totally not aware — Totally aware

0 25 50 75 100

Can Robyn take control to successfully deal with the situation?

Totally not — Totally

0 25 50 75 100

Supplementary figure 9. Vignette 4 (short distraction, unintentional distraction condition). Participants first read the vignette with a description of the situation and driver distraction, and then rate the driver's awareness, ability and finally attributed blame to the three actors (human driver, automated vehicle, and manufacturer.

In the same scenario:

> *Robyn is travelling along a curvy two-lane road at the speed limit with no traffic. Robocar is in full control of all the driving tasks and Robyn's only task is to supervise Robocar.*
>
> *The Robocar has been driving by itself for a long time. Robyn did not have to do any driving during this time. So, Robyn decides to read news articles on the vehicle's entertainment system. Because of this, Robyn is not paying attention to the Robocar and the road for a few minutes.*

Suddenly, Robocar encounters a tree branch in the middle of the lane and asks Robyn to take control immediately. Robyn does not successfully take over and an accident occurs.

To what extent is each actor responsible for the accident?

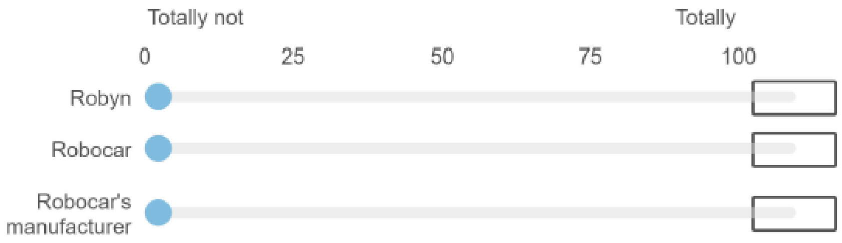

Supplementary figure 10. Vignette 4 (short distraction, unintentional distraction condition), continued. Participants first read the vignette with a description of the situation and driver distraction, and then rate the driver's awareness, ability and finally attributed blame to the three actors (human driver, automated vehicle, and manufacturer.

Vignette for condition 5: long distraction, intentional distraction

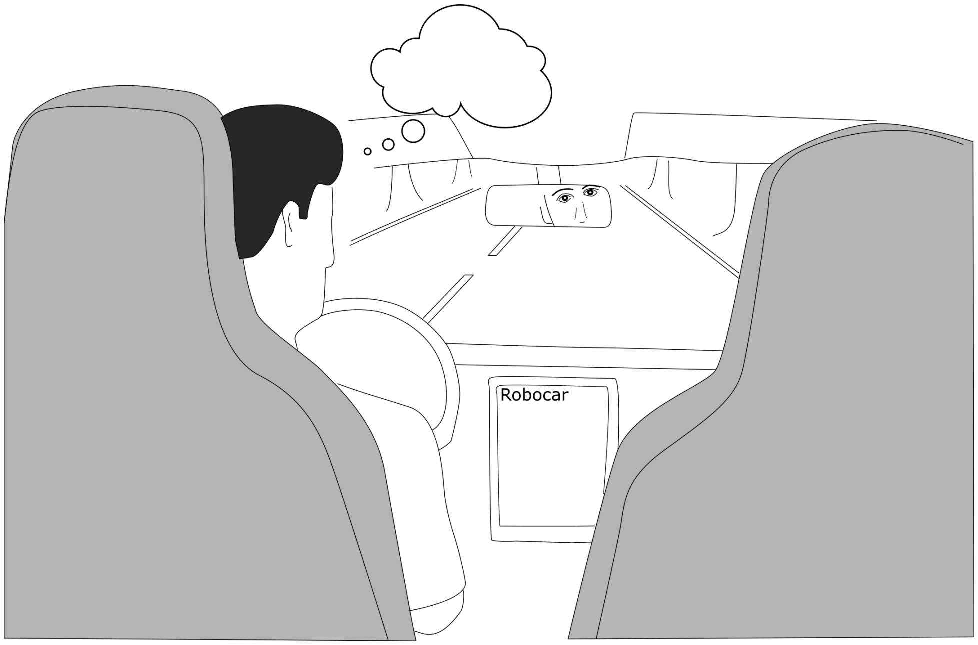

Robyn is travelling along a curvy two-lane road at the speed limit with no traffic. Robocar is in full control of all the driving tasks and Robyn's only task is to supervise Robocar.

The Robocar has been driving by itself for a long time. Robyn did not have to do any driving during this time. So, Robyn's mind completely wanders off to day-dream about holiday plans. Because of this, Robyn is not paying attention to the Robocar and the road for a few minutes.

Imagine that suddenly an unexpected situation happens on the road, for example a tree branch in the middle of the lane.

Supplementary figure 11. Vignette 5 (long distraction, intentional distraction condition). Participants first read the vignette with a description of the situation and driver distraction, and then rate the driver's awareness, ability and finally attributed blame to the three actors (human driver, automated vehicle, and manufacturer.

To what extent would Robyn be aware of the situation?

Totally not aware — Totally aware

0 25 50 75 100

Can Robyn take control to successfully deal with the situation?

Totally not — Totally

0 25 50 75 100

In the same scenario:

Robyn is travelling along a curvy two-lane road at the speed limit with no traffic. Robocar is in full control of all the driving tasks and Robyn's only task is to supervise Robocar.

The Robocar has been driving by itself for a long time. Robyn did not have to do any driving during this time. So, Robyn's mind completely wanders off to day-dream about holiday plans. Because of this, Robyn is not paying attention to the Robocar and the road for a few minutes.

Suddenly, Robocar encounters a tree branch in the middle of the lane and asks Robyn to take control immediately. Robyn does not successfully take over and an accident occurs.

To what extent is each actor responsible for the accident?

Totally not — Totally

0 25 50 75 100

Robyn

Robocar

Robocar's manufacturer

Supplementary figure 12. Vignette 5 (long distraction, intentional distraction condition), continued. Participants first read the vignette with a description of the situation and driver distraction, and then rate the driver's awareness, ability and finally attributed blame to the three actors (human driver, automated vehicle, and manufacturer.

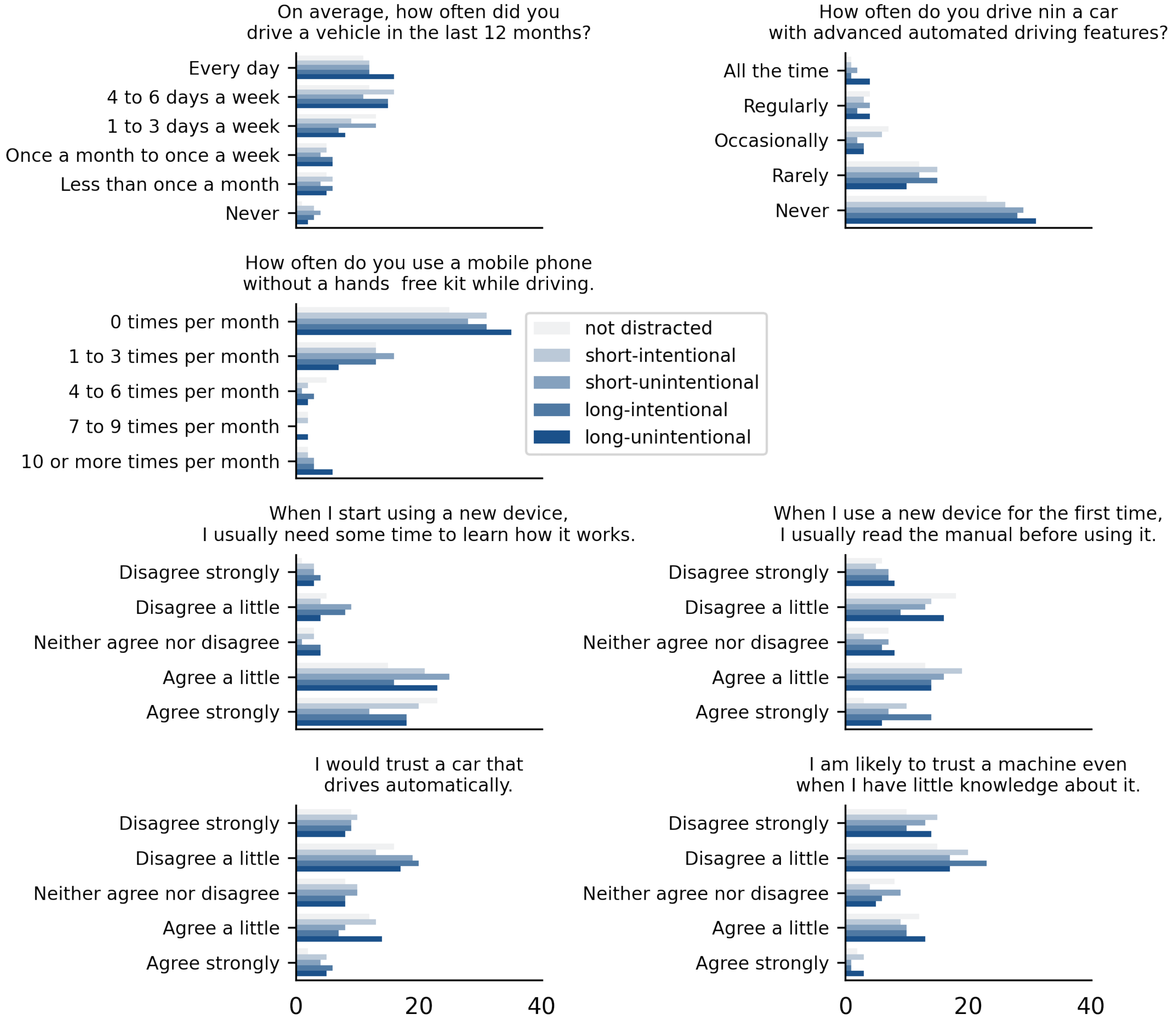

Supplementary figure 13. Participant answers with respect to driving experience, general attitudes towards driving automation, mobile phone use, technology adoption, and trust towards driving automation. Answers are split per scenario. Participant answers are relatively evenly distributed over scenarios. Participants who preferred not to respond were excluded from this analysis.

Thematic analysis

Supplementary figures 14 to 17 show the results of the thematic analysis. The results are split per theme" responsibility primarily to the driver, to the vehicle, and to the manufacturer, respectively. The figures show the codes, subcodes, and a quote per subcode.

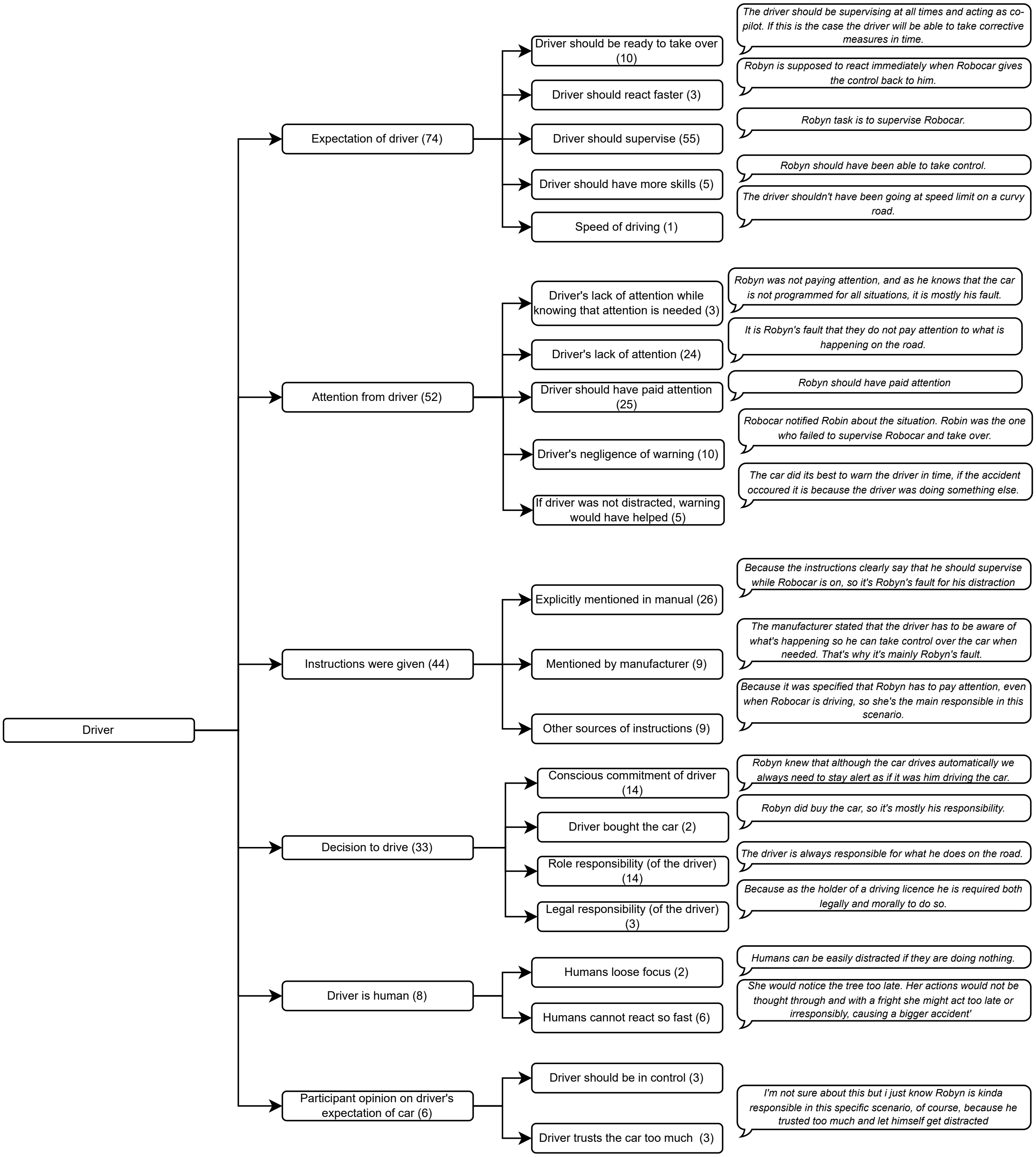

Supplementary figure 14. Codes, subcodes, and representative quote per subcode, with arguments attributing responsibility toward the driver.

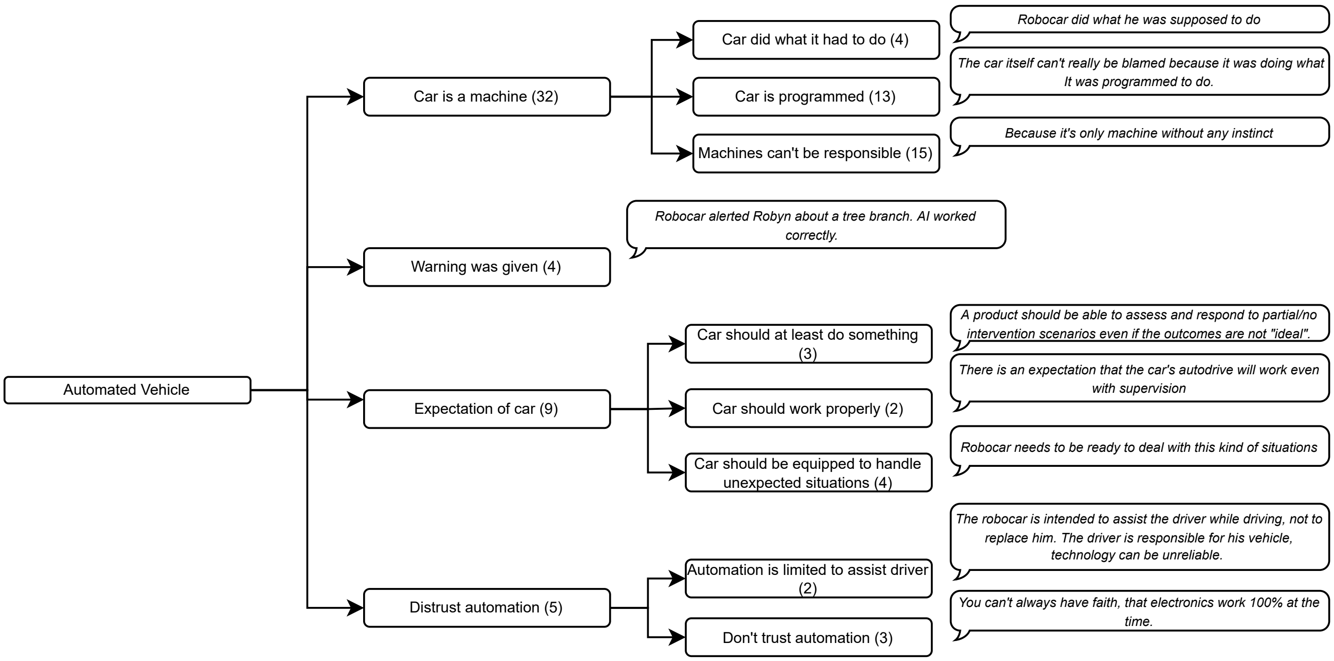

Supplementary figure 15. Codes, subcodes, and representative quote per subcode, with arguments attributing responsibility toward the automated vehicle. Following the scenario descriptions, some participants referred to the driver as 'Robyn' and the vehicle as 'Robocar'.

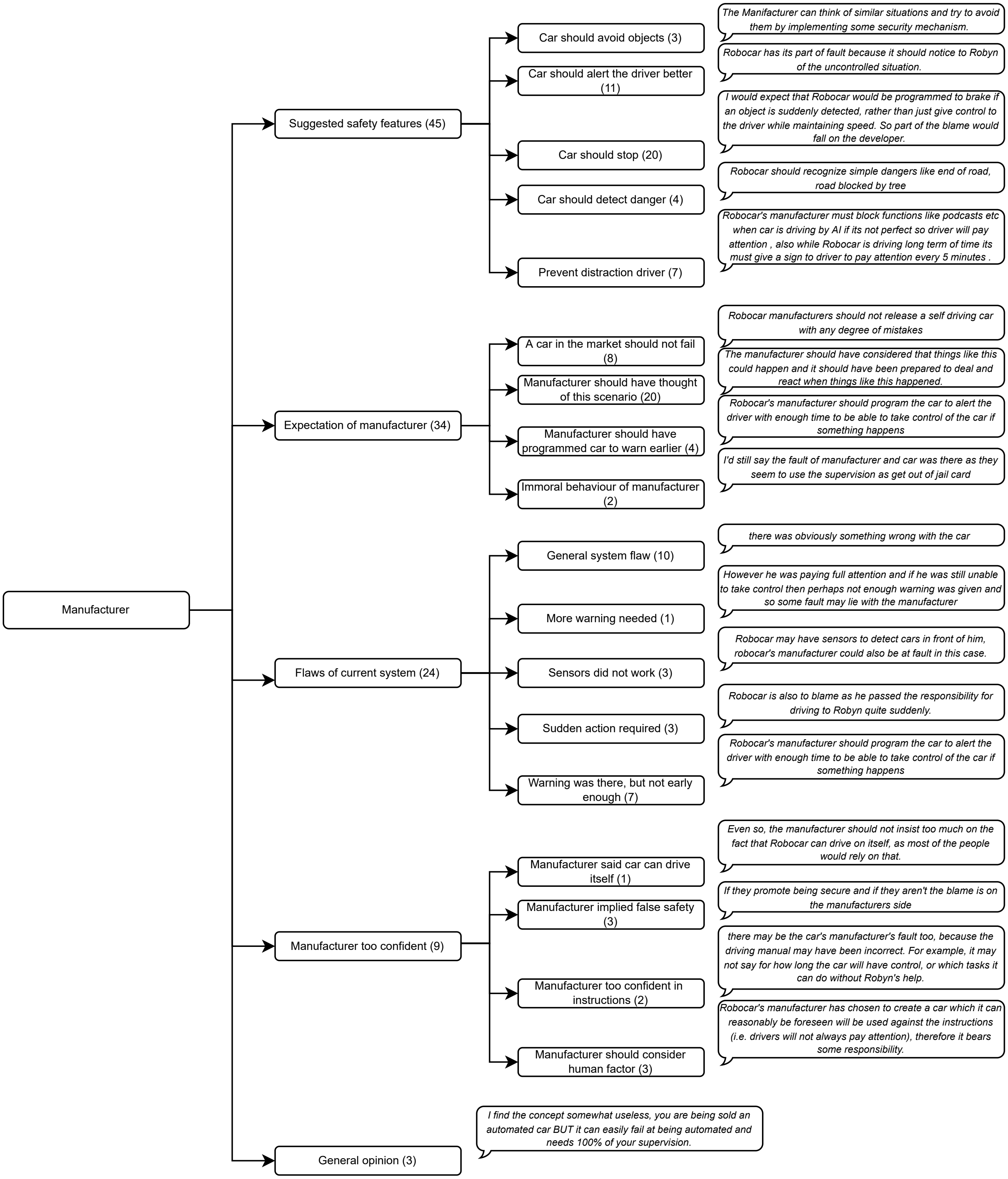

Supplementary figure 16. Codes, subcodes, and representative quote per subcode, with arguments attributing responsibility toward the manufacturer. Following the scenario descriptions, some participants referred to the driver as 'Robyn' and the vehicle as 'Robocar'.

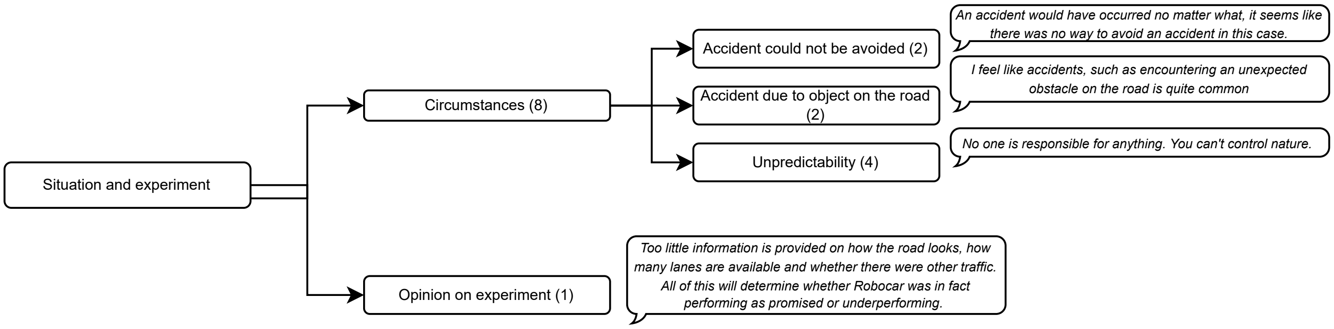

Supplementary figure 17. Codes, subcodes, and representative quote per subcode, with arguments attributing responsibility concerning the situation or the experiment itself. Following the scenario descriptions, some participants referred to the driver as 'Robyn' and the vehicle as 'Robocar'.

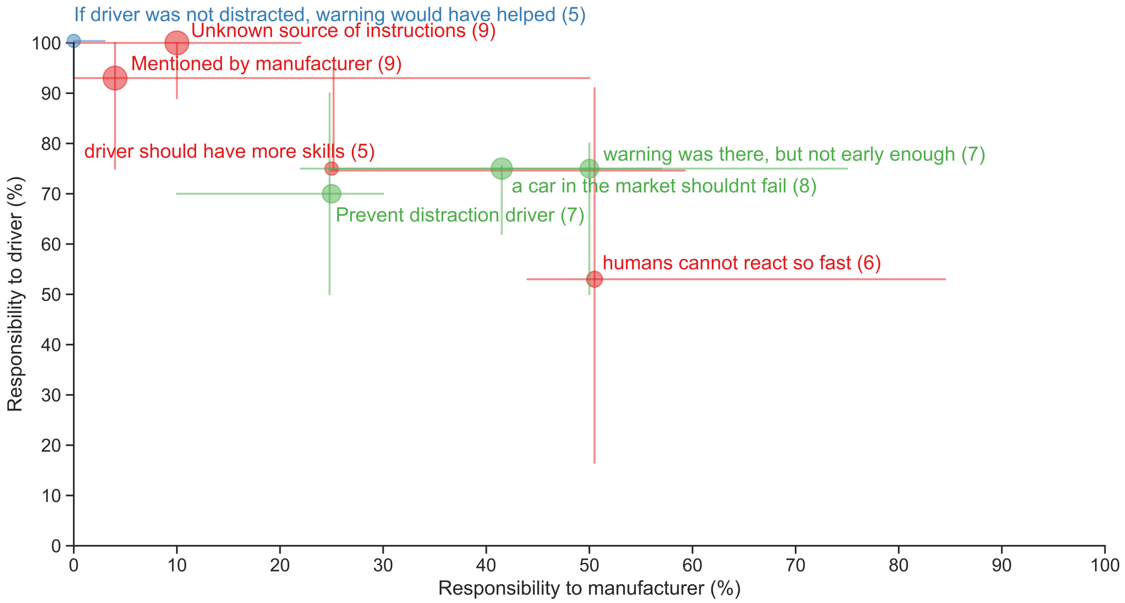

Supplementary figure 18. Median responsibility attribution to the driver and the manufacturer per code identified in the thematic analysis of the participants' reasoning. Codes that were mentioned between 5 and 10 times are shown, in addition to Fig. 4. The number of times the argument was made is included in brackets. The lines indicate 95% confidence interval of the median responsibility attributions for each code.

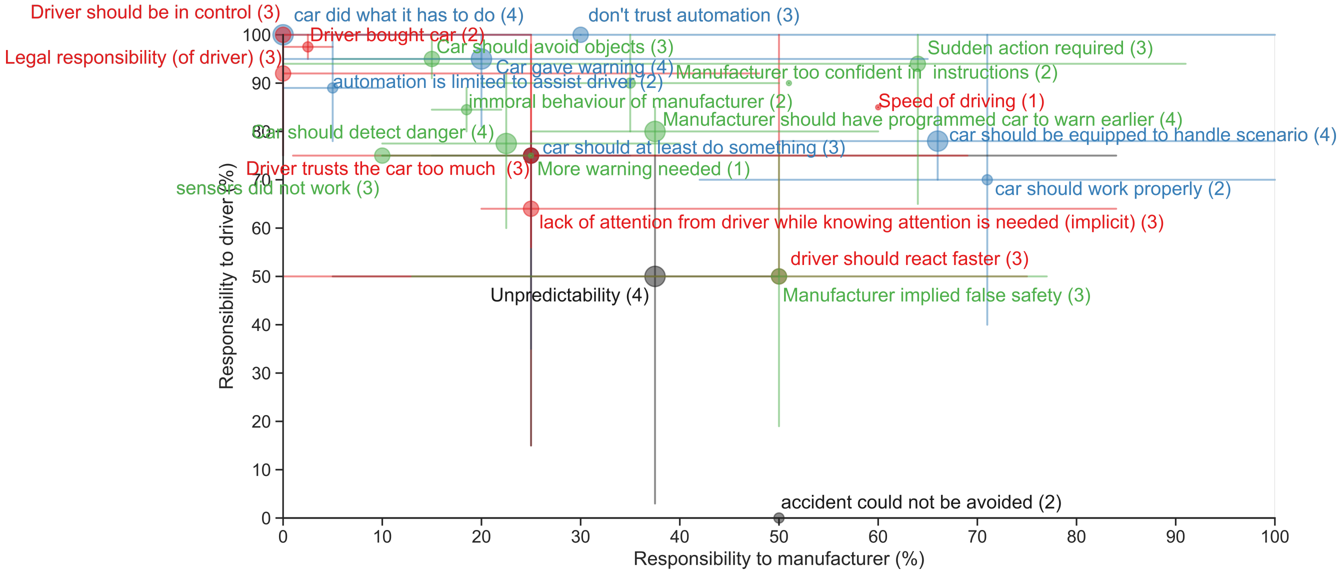

Supplementary figure 19. Median responsibility attribution to the driver and the manufacturer per code identified in the thematic analysis of the participants' reasoning. Codes that were mentioned less than 5 times are shown, in addition to Fig. 4. The number of times the argument was made is included in brackets. The lines indicate 95% confidence interval of the median responsibility attributions for each code.

Supplementary Table 2. Number of times a code was mentioned as argument for the intentional and unintentional scenarios.

Code	Intentional	Unintentional
Expectation of the driver	31	25
Attention of the driver	23	25
Instructions given	19	21
Suggested safety features	17	20
Decision to drive	11	14
Expectation of the manufacturer	8	18
Car is machine	7	19
Flaws of the current system	7	6
Warning was given	5	7
Manufacturer is too confident	5	4
Distrust automation	3	1
Circumstances	2	-
Driver is human	2	4
Expectation of the car	2	4
Participant opinion on driver's expectation of car	1	4
Opinion on experiment	1	-
General opinion	-	3
